# Supplementary material for: Human genetic adaptation related to cellular zinc homeostasis
Source: PLoS Genet. 2023 Sep 25;19(9):e1010950. doi: 10.1371/journal.pgen.1010950 (PMC10553801; doi:10.1371/journal.pgen.1010950)
Supplement: S1 Fig — (A) Allele frequencies at rs1047626 and rs4861157 across human populations in the 1000 Genomes Project [63]. Frequency plots were downloaded from the Geography of Genetic Variants Browser (https://popgen.uchicago.edu/ggv/) [73] (B) Differential SLC30A9 expression in the substantia nigra according to the rs1047626 and rs4861157 genotypes as available at the GTEX portal (https://www.gtexportal.org/home/). NES, normalized effect sizes. (PDF) [file pgen.1010950.s001.pdf]

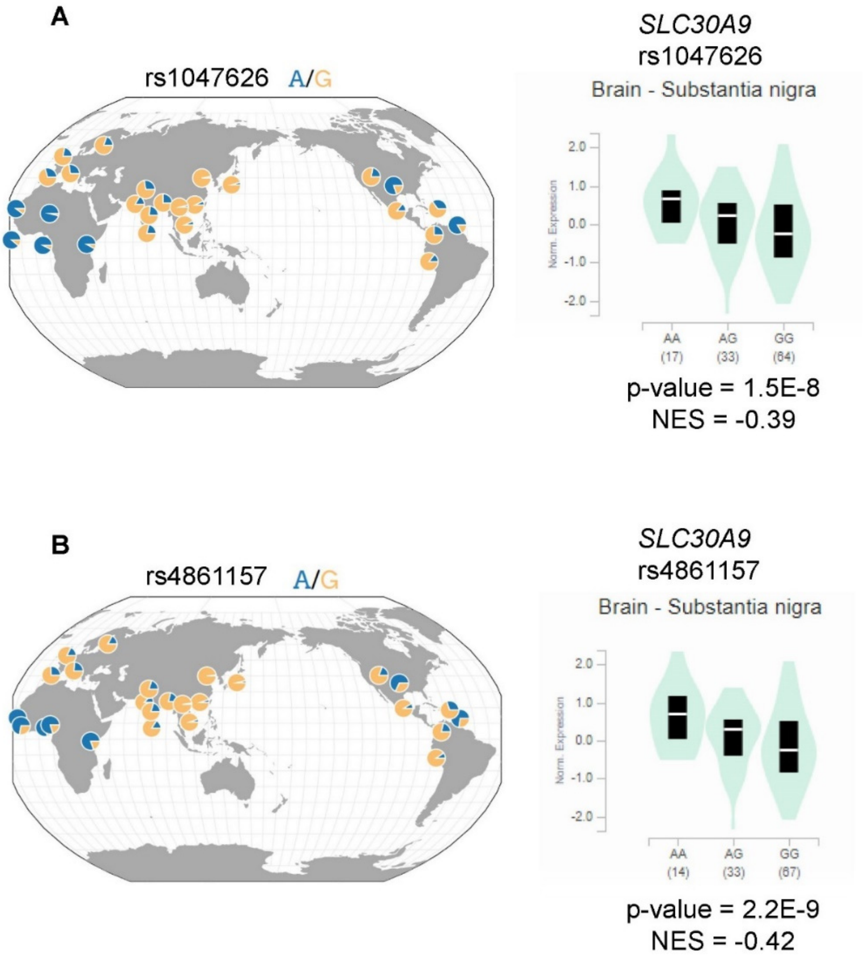
A
rs1047626 A/G
SLC30A9
rs1047626
Brain - Substantia nigra
Norm. Expression
2.0
1.0
0.0
-1.0
-2.0
AA
(17)
AG
(33)
GG
(64)
p-value = 1.5E-8
NES = -0.39
B
rs4861157 A/G
SLC30A9
rs4861157
Brain - Substantia nigra
Norm. Expression
2.0
1.0
0.0
-1.0
-2.0
AA
(14)
AG
(33)
GG
(67)
p-value = 2.2E-9
NES = -0.42
