# Supplementary figures and images for: Human genetic adaptation related to cellular zinc homeostasis
Source: PLoS Genet. 2023 Sep 25;19(9):e1010950. doi: 10.1371/journal.pgen.1010950 (PMC10553801; doi:10.1371/journal.pgen.1010950)

A

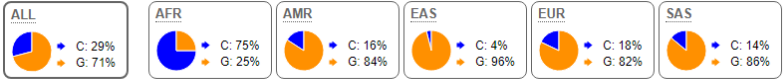

B

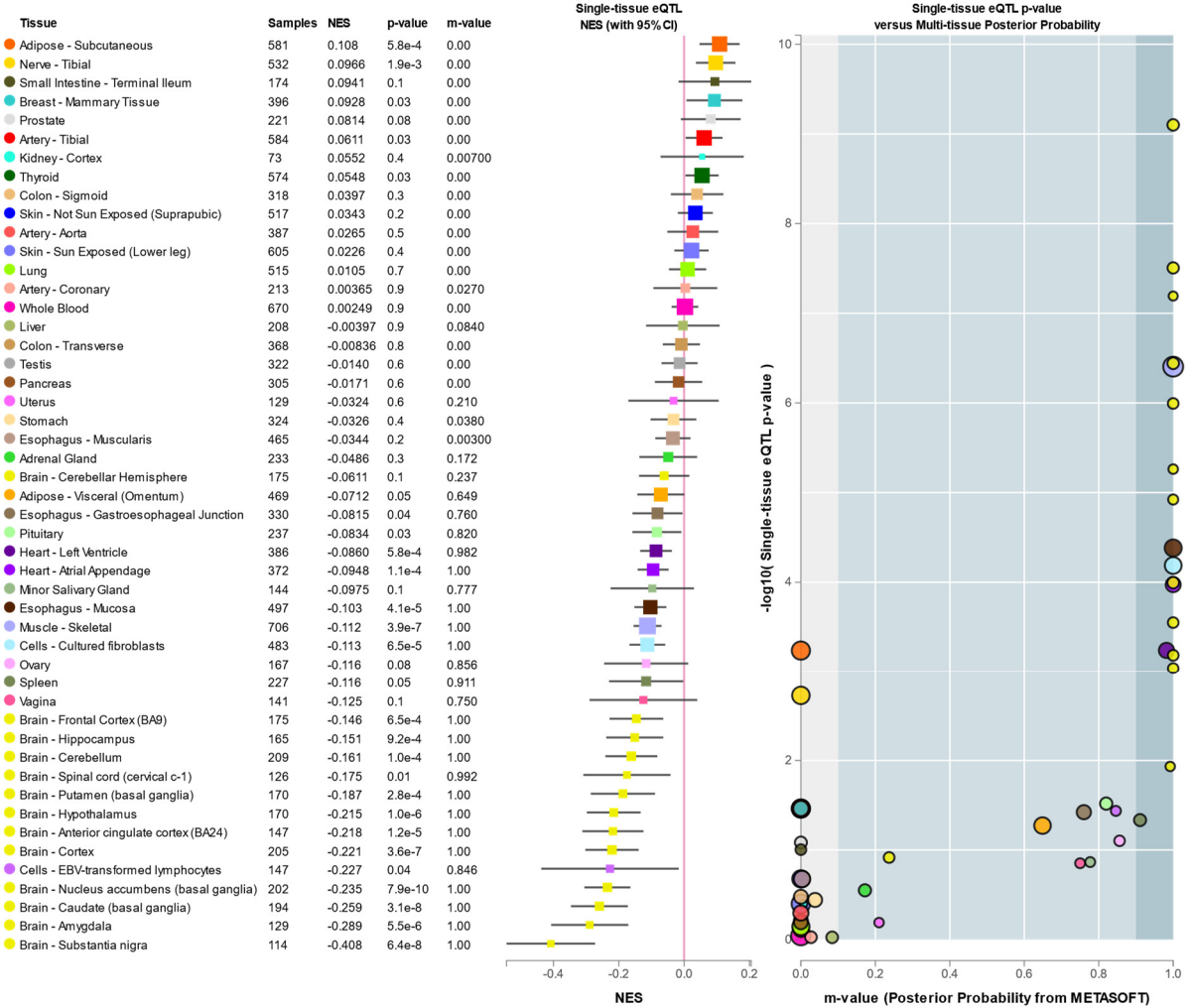

C

SLC30A9  
chr4\_41990660\_C\_G\_b38  
Brain - Nucleus accumbens (basal ganglia)

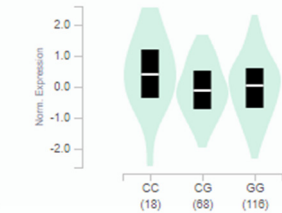

Supplement: S2 Fig — (A) Continental 1000 Genomes Project Phase 3 allele frequencies as retrieved from Ensembl (https://www.ensembl.org/index.html). (B) Multi-tissue eQTL comparison for rs2581434. (C). Differential SLC30A9 expression in the substantia nigra according to the rs2581434 genotypes as available at the GTEX portal (https://www.gtexportal.org/home/). NES, normalized effect sizes. (PDF) [file pgen.1010950.s002.pdf]

A

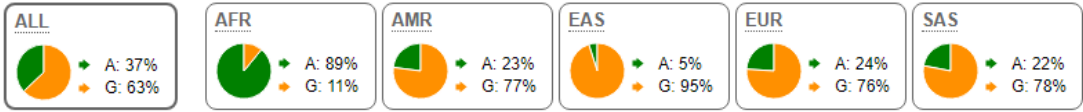

B

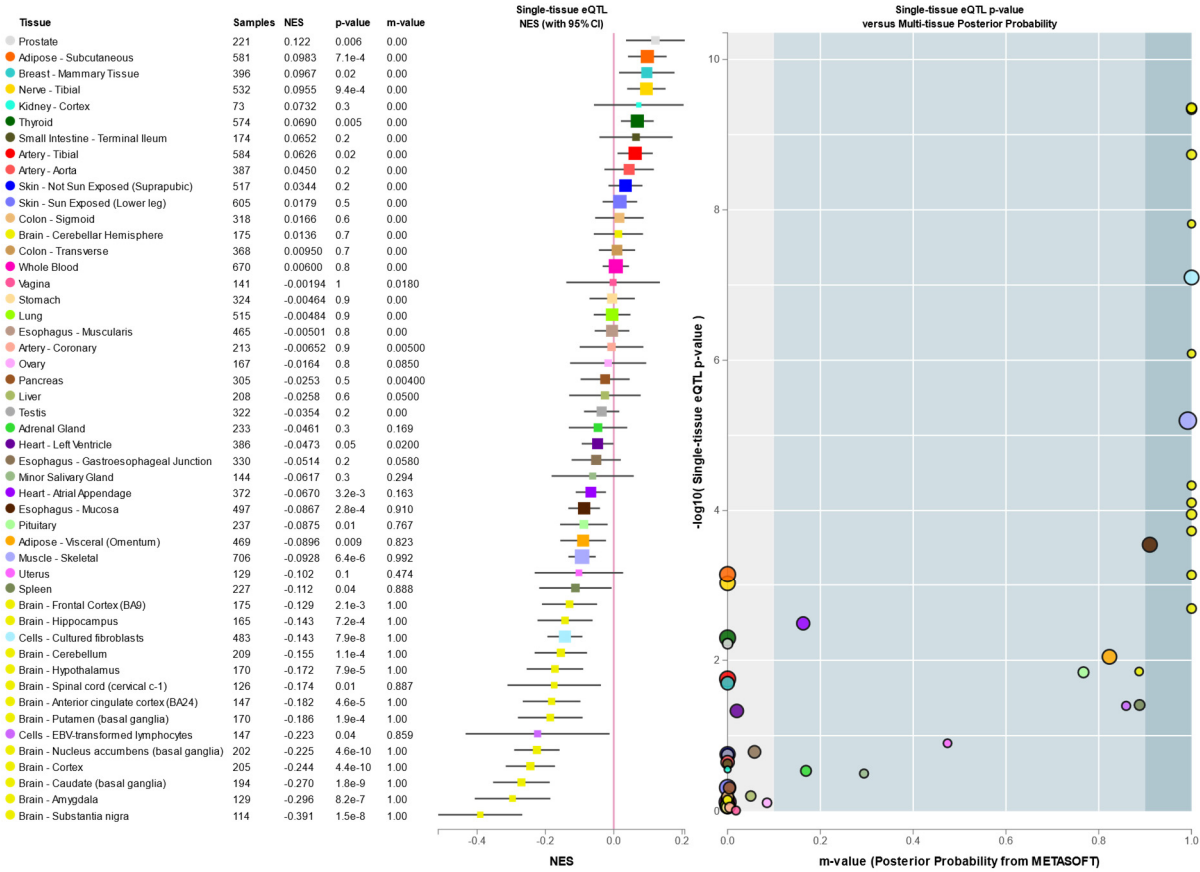

C

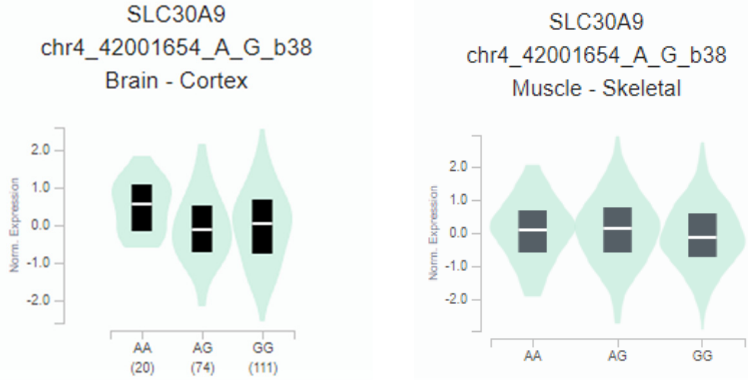

Supplement: S3 Fig — (A) Continental 1000 Genomes Project Phase 3 allele frequencies as retrieved from Ensembl (https://www.ensembl.org/index.html). (B) Multi-tissue eQTL comparison for rs1047626. (C). Differential SLC30A9 expression in brain cortex and skeletal muscle according to the rs1047626 genotypes as available at the GTEX portal (https://www.gtexportal.org/home/). NES, normalized effect sizes. (PDF) [file pgen.1010950.s003.pdf]

A

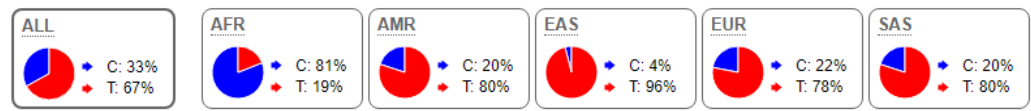

B

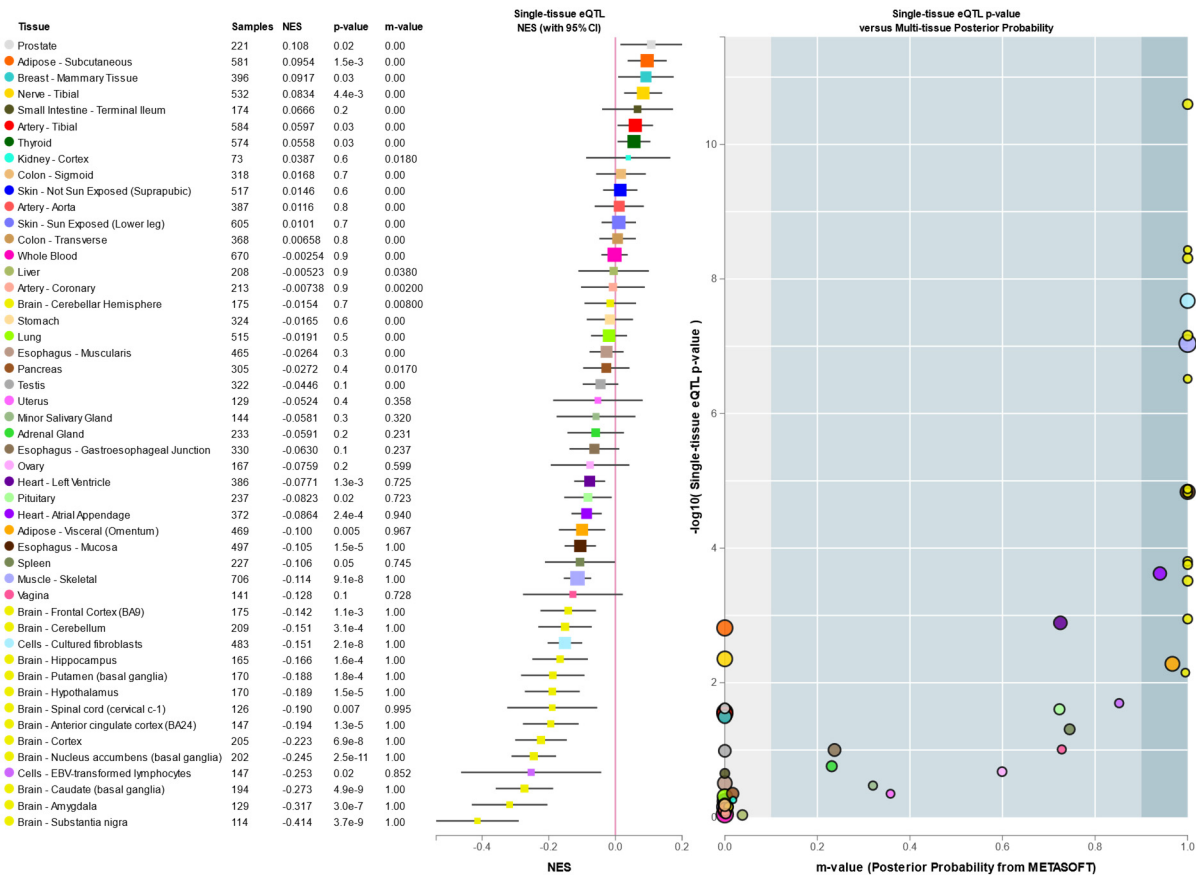

C

SLC30A9  
chr4\_42002961\_C\_T\_b38  
Brain - Nucleus accumbens (basal ganglia)

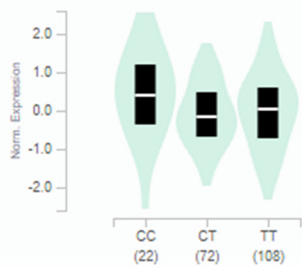

Supplement: S4 Fig — (A) Continental 1000 Genomes Project Phase 3 allele frequencies as retrieved from Ensembl (https://www.ensembl.org/index.html). (B) Multi-tissue eQTL comparison for rs2581452. (C). Differential SLC30A9 expression in the nucleus accumbens according to the rs2581452 genotypes as available at the GTEX portal (https://www.gtexportal.org/home/). NES, normalized effect sizes. (PDF) [file pgen.1010950.s004.pdf]

A

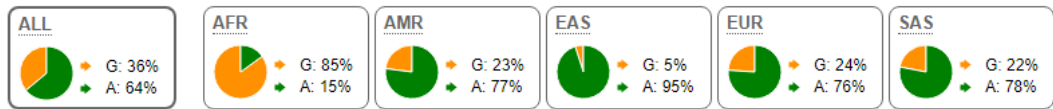

B

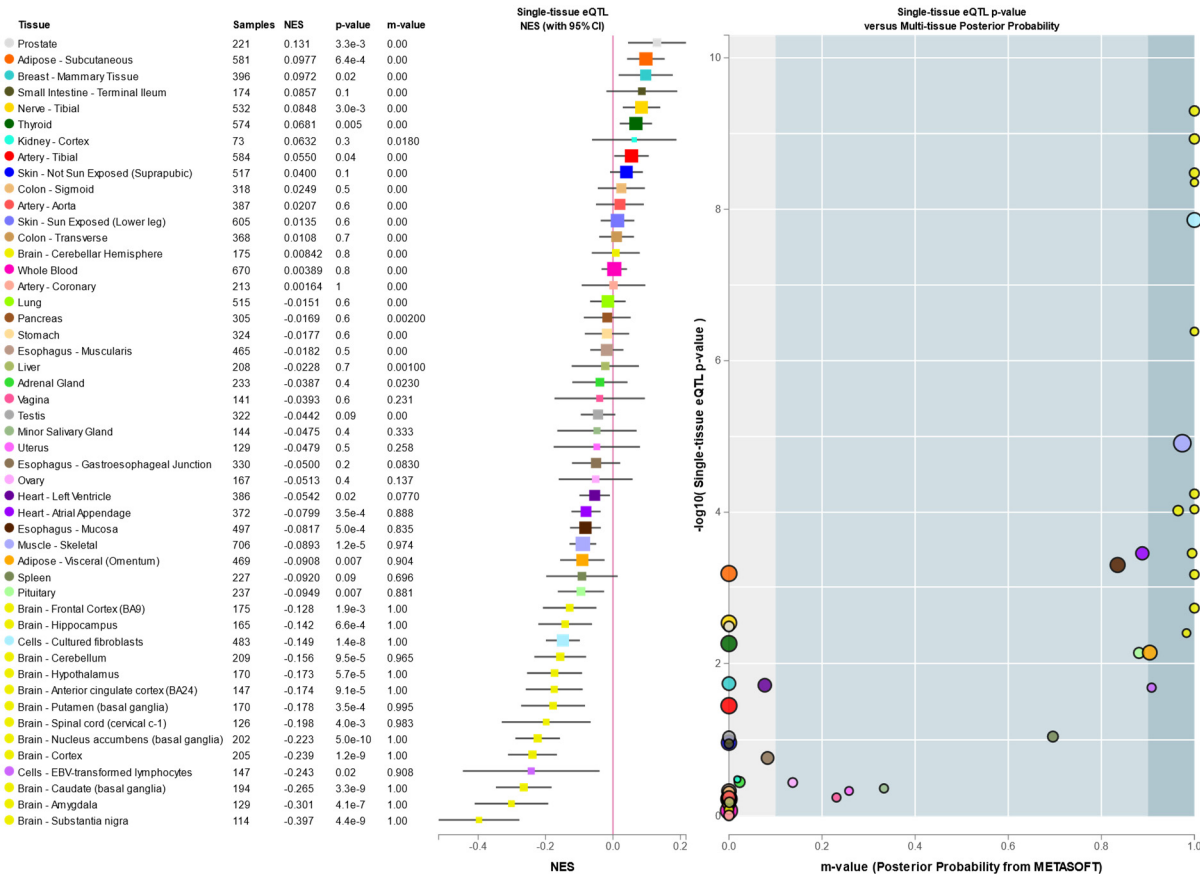

C

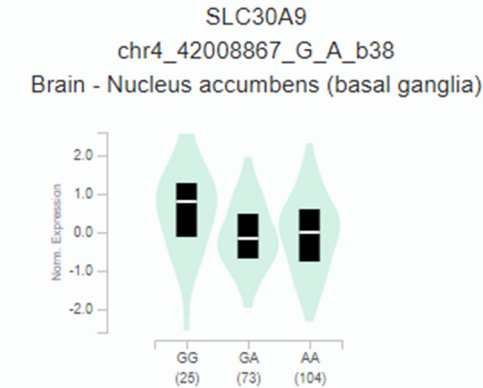

Supplement: S5 Fig — (A) Continental 1000 Genomes Project Phase 3 allele frequencies as retrieved from Ensembl (https://www.ensembl.org/index.html). (B) Multi-tissue eQTL comparison for rs2660319. (C). Differential SLC30A9 expression in the nucleus accumbens according to the rs2660319 genotypes as available at the GTEX portal (https://www.gtexportal.org/home/). NES, normalized effect sizes. (PDF) [file pgen.1010950.s005.pdf]

A

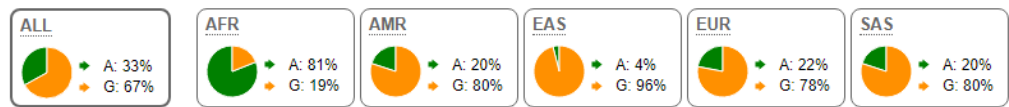

B

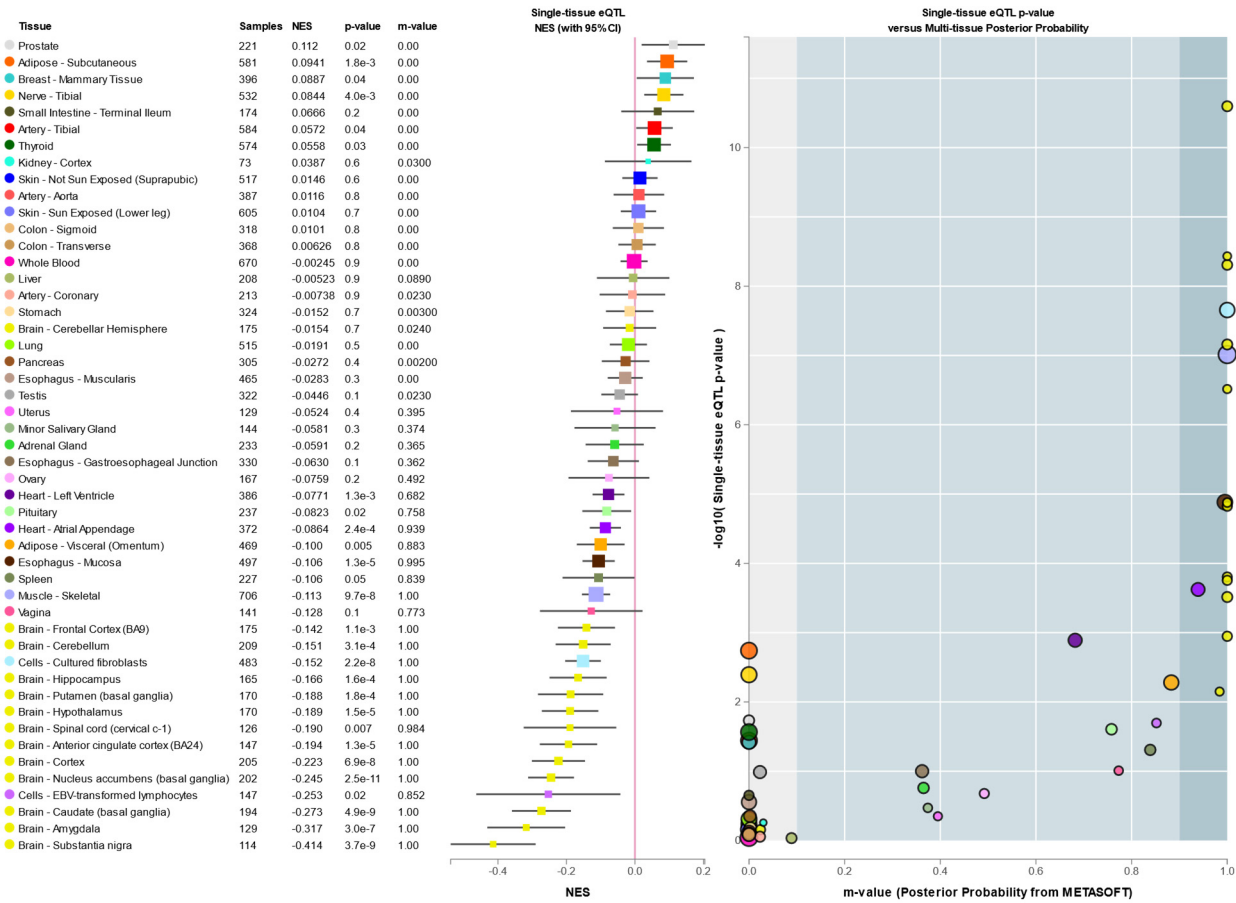

C

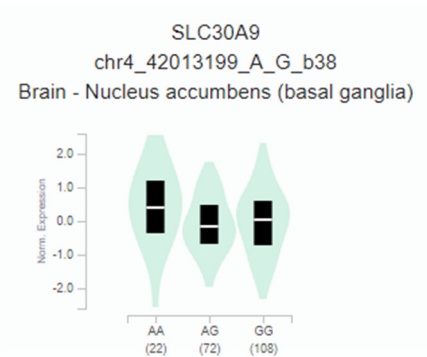

Supplement: S6 Fig — (A) Continental 1000 Genomes Project Phase 3 allele frequencies as retrieved from Ensembl (https://www.ensembl.org/index.html). (B) Multi-tissue eQTL comparison for rs1848182. (C). Differential SLC30A9 expression in the nucleus accumbens according to the rs1848182 genotypes as available at the GTEX portal (https://www.gtexportal.org/home/). NES, normalized effect sizes. (PDF) [file pgen.1010950.s006.pdf]

A

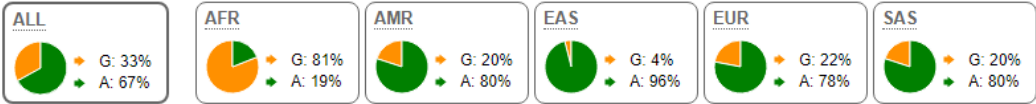

B

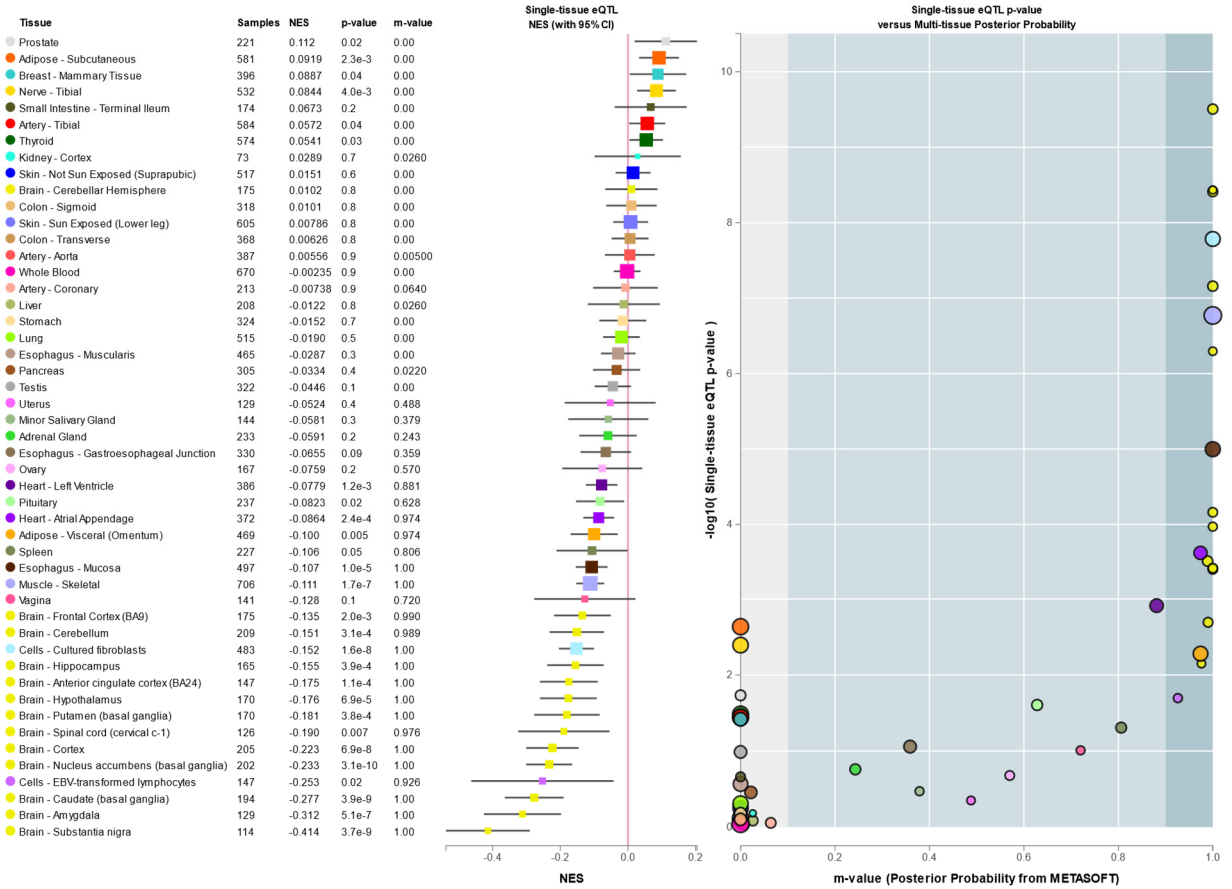

C

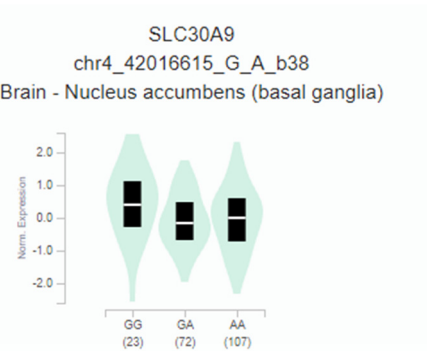

Supplement: S7 Fig — (A) Continental 1000 Genomes Project Phase 3 allele frequencies as retrieved from Ensembl (https://www.ensembl.org/index.html). (B) Multi-tissue eQTL comparison for rs2581424. (C). Differential SLC30A9 expression in the nucleus accumbens according to the rs2581424 genotypes as available at the GTEX portal (https://www.gtexportal.org/home/). NES, normalized effect sizes. (PDF) [file pgen.1010950.s007.pdf]

A

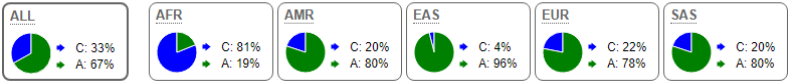

B

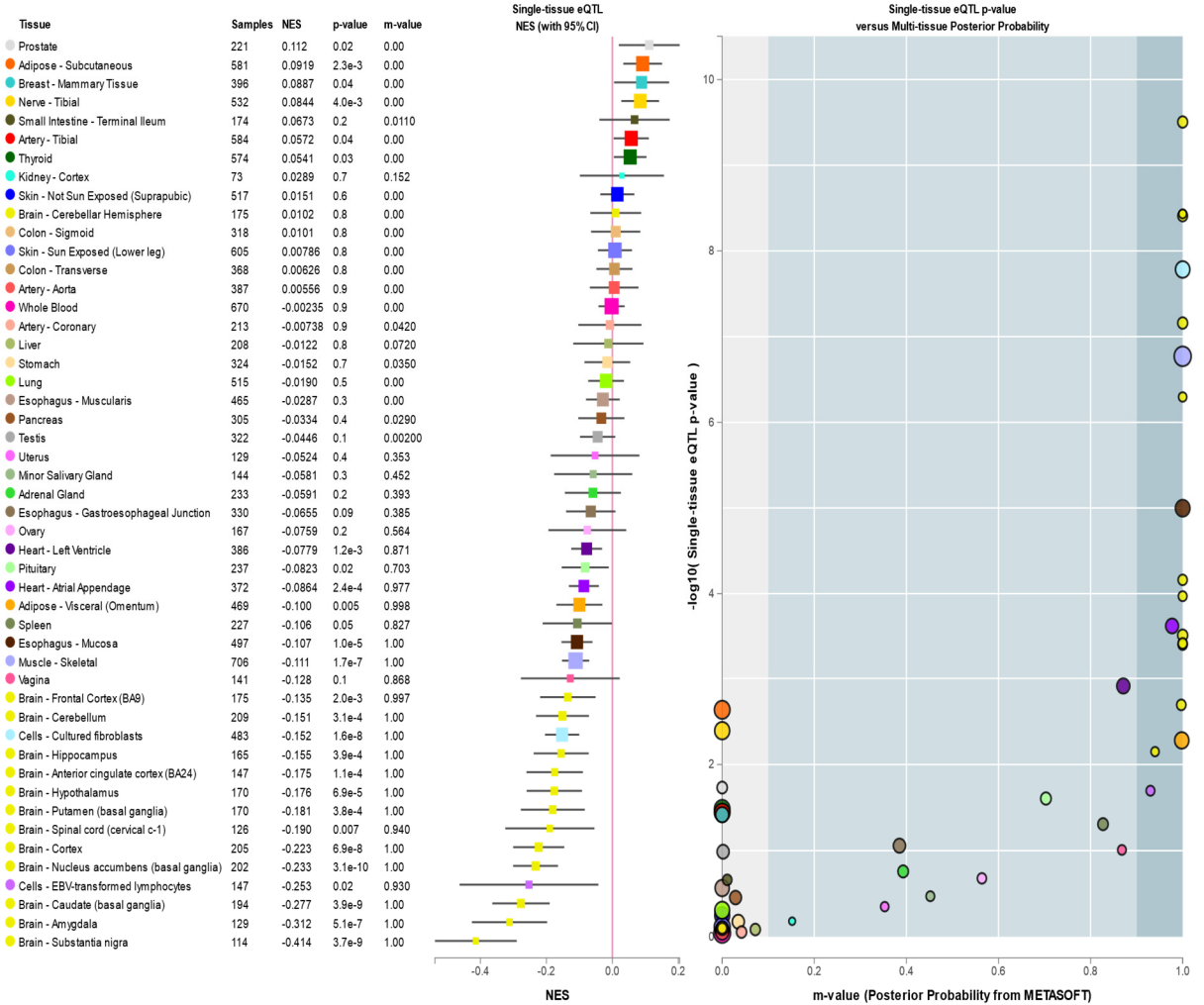

C

SLC30A9  
chr4\_42020447\_C\_A\_b38  
Brain - Nucleus accumbens (basal ganglia)

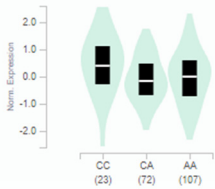

Supplement: S8 Fig — (A) Continental 1000 Genomes Project Phase 3 allele frequencies as retrieved from Ensembl (https://www.ensembl.org/index.html). (B) Multi-tissue eQTL comparison for rs15857. (C). Differential SLC30A9 expression in the nucleus accumbens according to the rs15857 genotypes as available at the GTEX portal (https://www.gtexportal.org/home/). NES, normalized effect sizes. (PDF) [file pgen.1010950.s008.pdf]

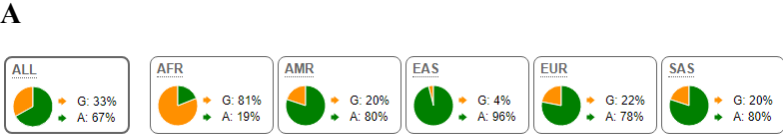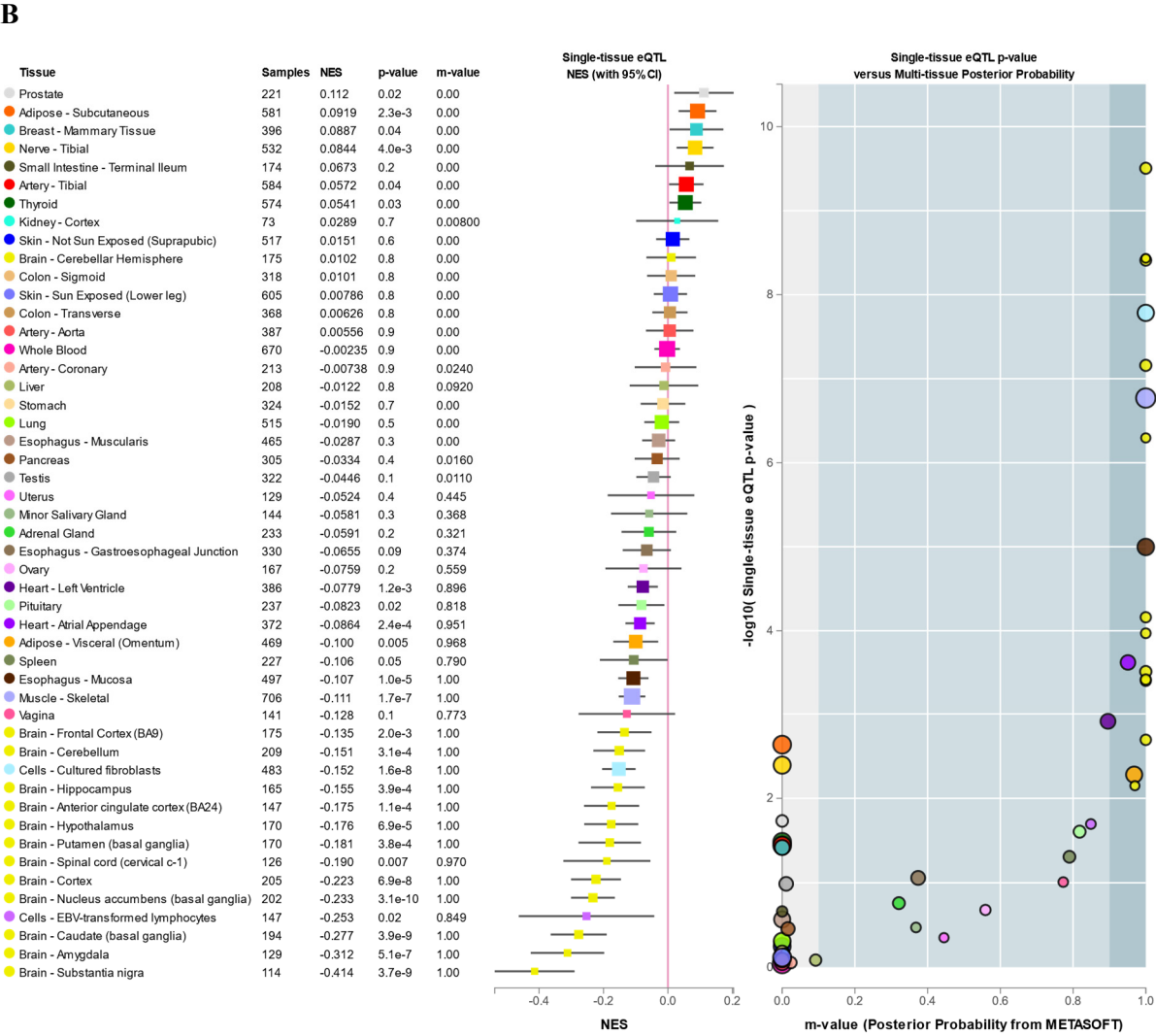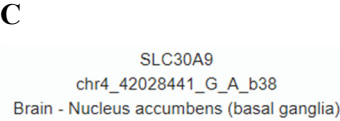

Supplement: S9 Fig — (A) Continental 1000 Genomes Project Phase 3 allele frequencies as retrieved from Ensembl (https://www.ensembl.org/index.html). (B) Multi-tissue eQTL comparison for rs7439806. (C). Differential SLC30A9 expression in the nucleus accumbens according to the rs7439806 genotypes as available at the GTEX portal (https://www.gtexportal.org/home/). NES, normalized effect sizes. (PDF) [file pgen.1010950.s009.pdf]

A

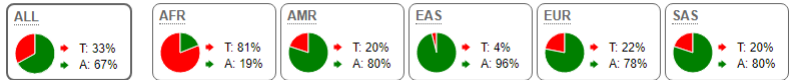

B

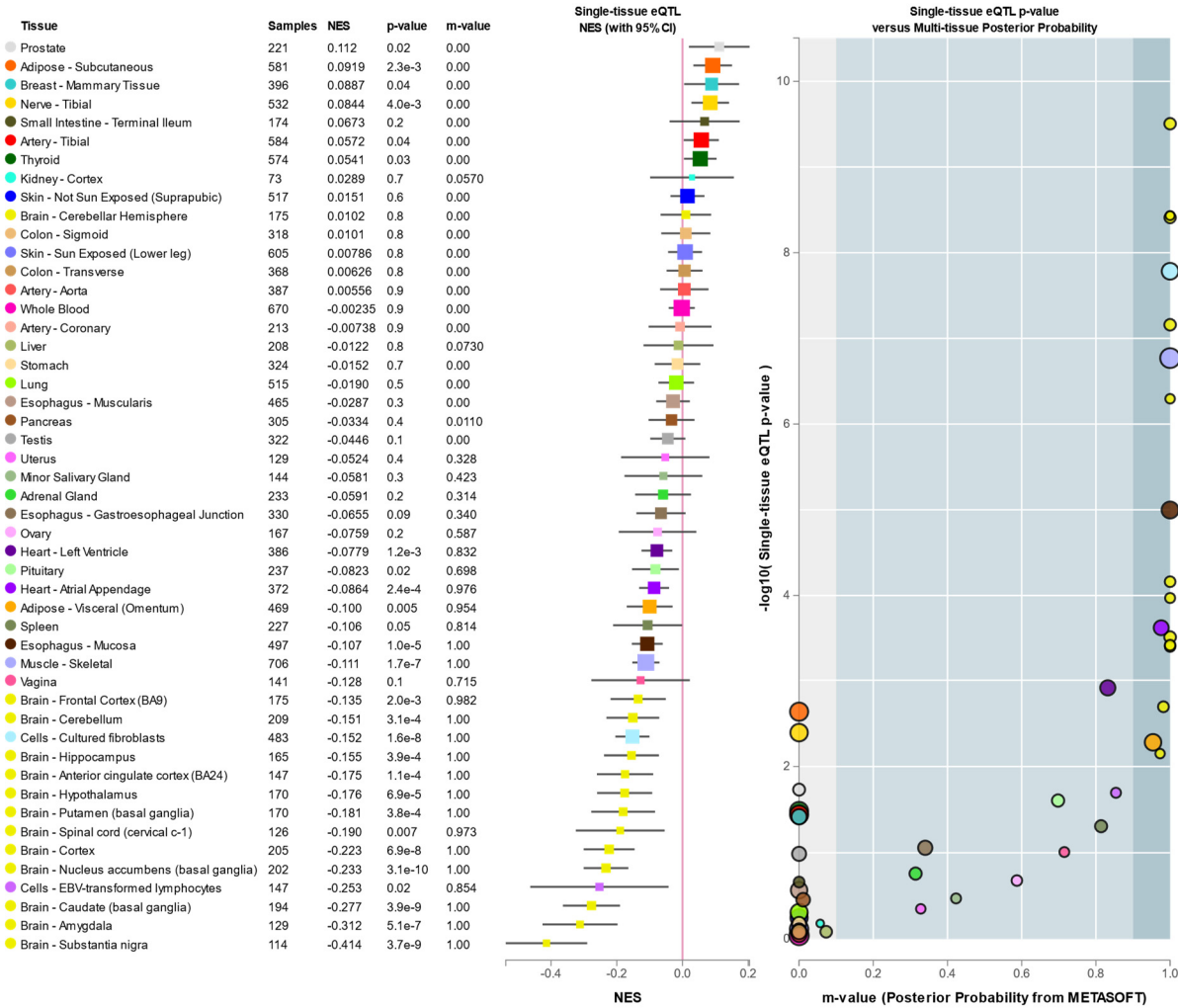

C

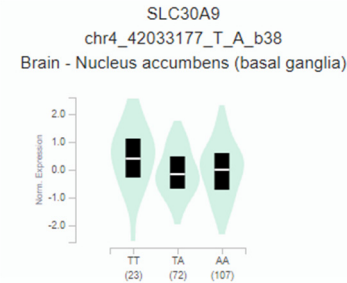

Supplement: S10 Fig — (A) Continental 1000 Genomes Project Phase 3 allele frequencies as retrieved from Ensembl (https://www.ensembl.org/index.html). (B) Multi-tissue eQTL comparison for rs55835604. (C). Differential SLC30A9 expression in the nucleus accumbens according to the rs55835604 genotypes as available at the GTEX portal (https://www.gtexportal.org/home/). NES, normalized effect sizes. (PDF) [file pgen.1010950.s010.pdf]

A

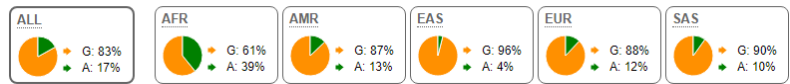

B

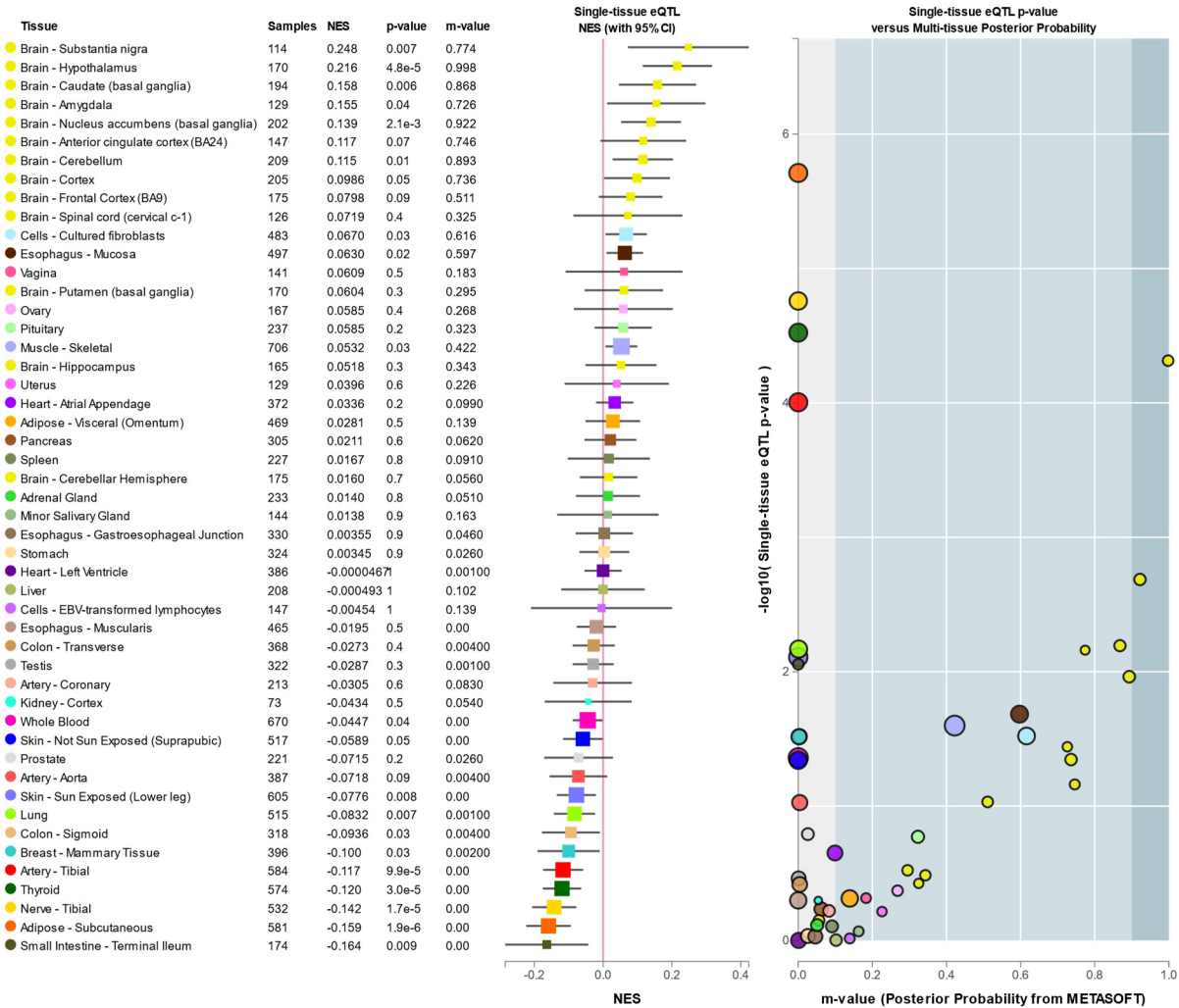

C

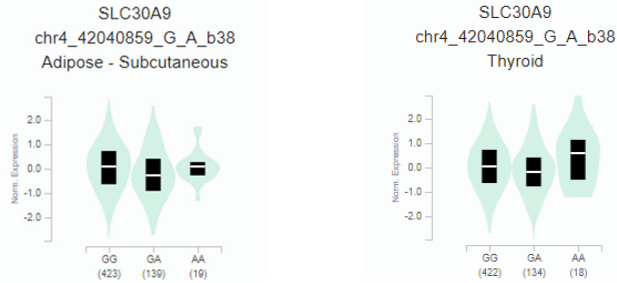

Supplement: S11 Fig — (A) Continental 1000 Genomes Project Phase 3 allele frequencies as retrieved from Ensembl. (B) Multi-tissue eQTL comparison for rs12510574. (C) Differential SLC30A9 expression in the subcutaneous adipose tissue and the thyroid according to the rs12510574 genotypes as available at the GTEX portal (https://www.gtexportal.org/home/). NES, normalized effect sizes. (PDF) [file pgen.1010950.s011.pdf]

A

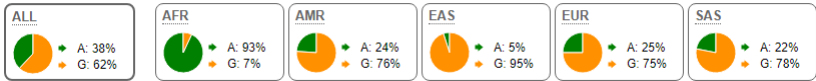

B

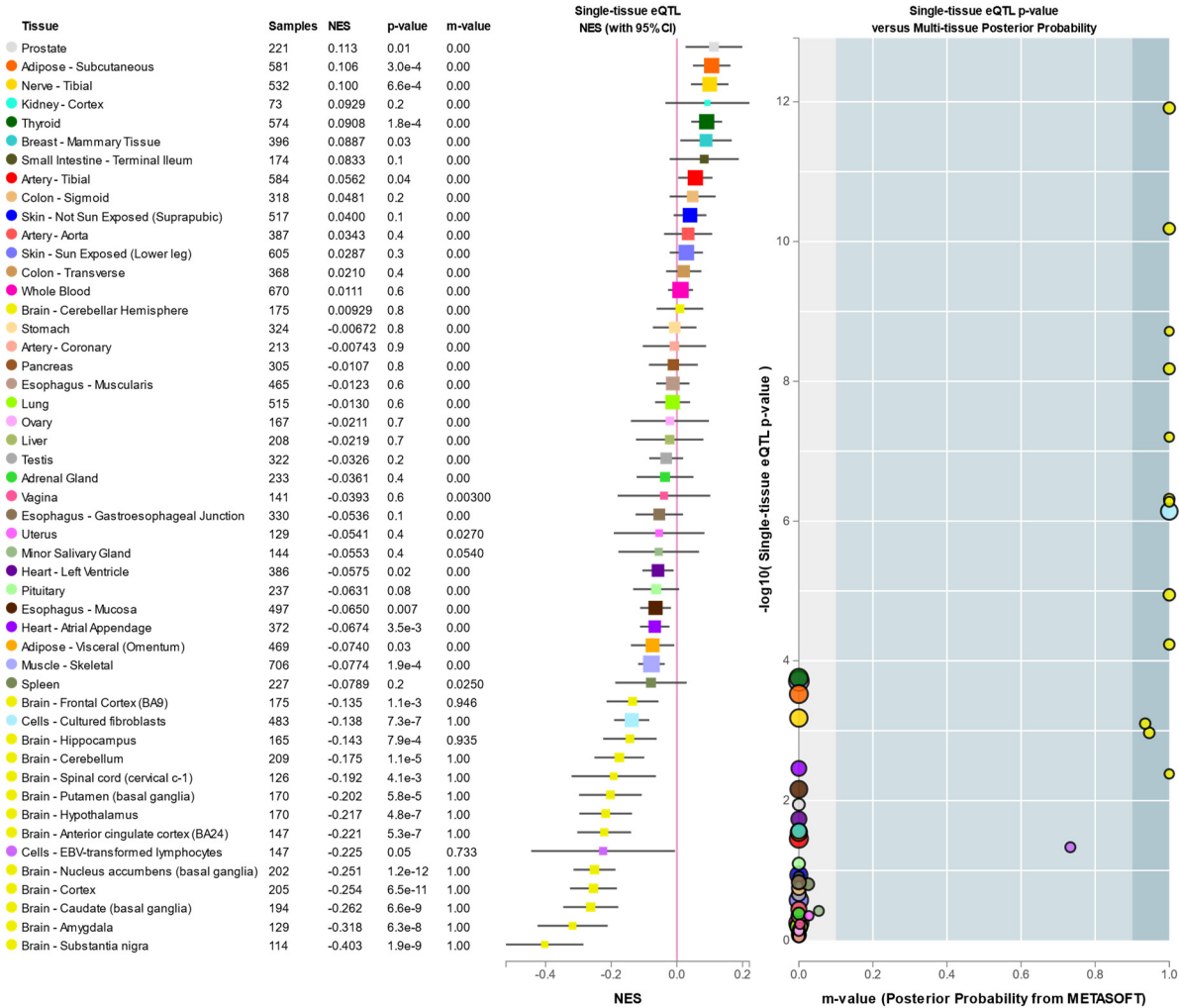

C

SLC30A9  
chr4\_42055122\_A\_G\_b38  
Brain - Nucleus accumbens (basal ganglia)

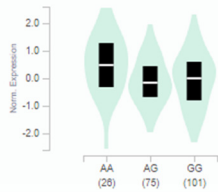

Supplement: S12 Fig — (A) Continental 1000 Genomes Project Phase 3 allele frequencies as retrieved from Ensembl (https://www.ensembl.org/index.html). (B) Multi-tissue eQTL comparison for rs4861014. (C). Differential SLC30A9 expression in the nucleus accumbens according to the rs4861014 genotypes as available at the GTEX portal (https://www.gtexportal.org/home/). NES, normalized effect sizes. (PDF) [file pgen.1010950.s012.pdf]

A

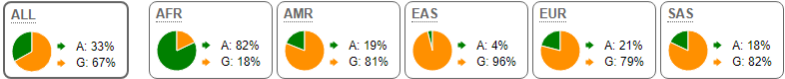

B

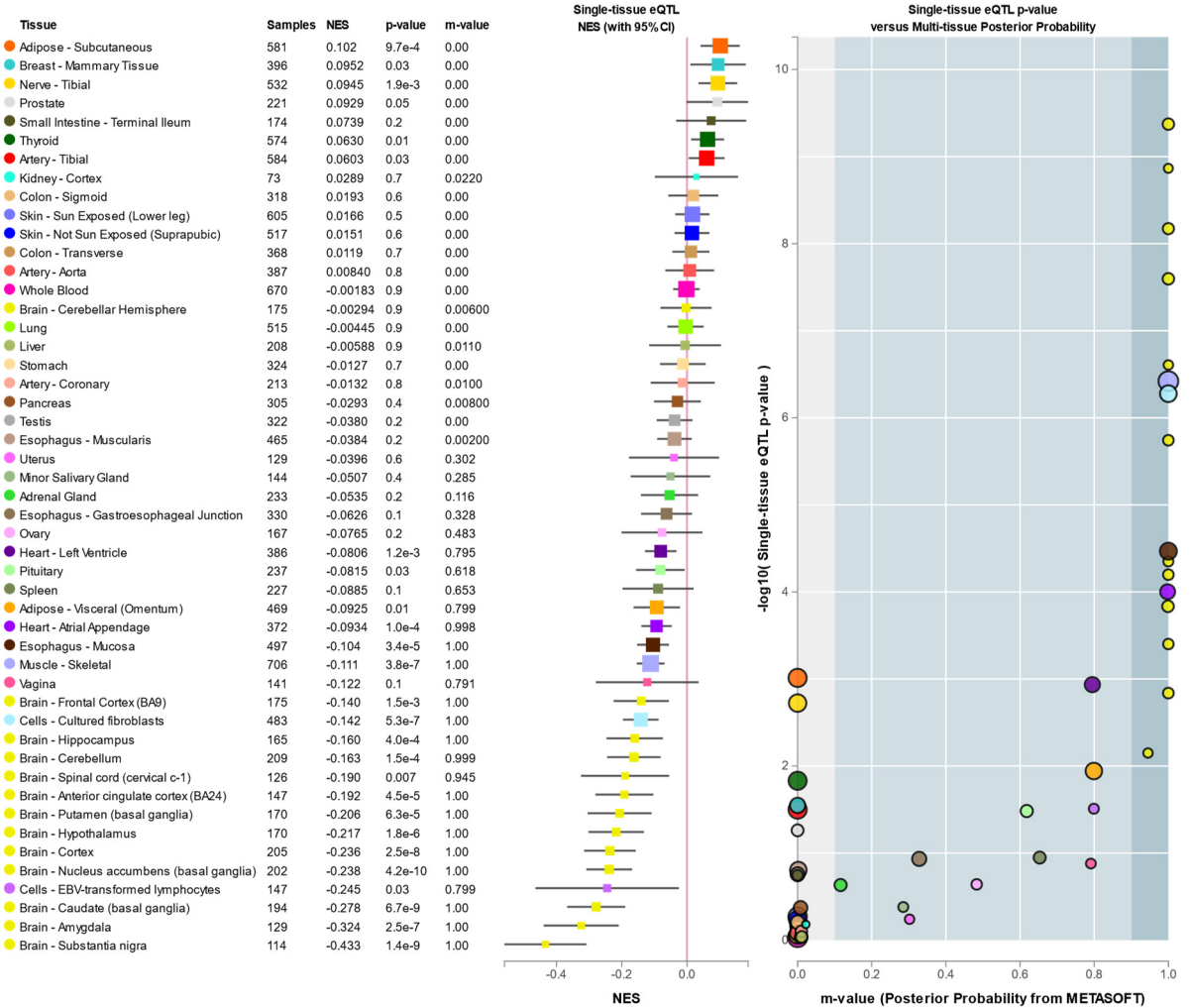

C

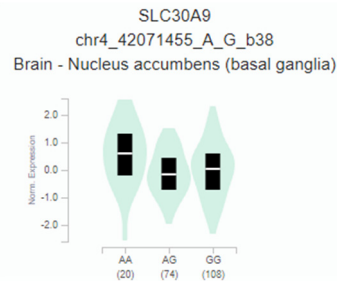

Supplement: S13 Fig — (A) Continental 1000 Genomes Project Phase 3 allele frequencies as retrieved from Ensembl (https://www.ensembl.org/index.html). (B) Multi-tissue eQTL comparison for rs10019356. (C). Differential SLC30A9 expression in the nucleus accumbens according to the rs10019356 genotypes as available at the GTEX portal (https://www.gtexportal.org/home/). NES, normalized effect sizes. (PDF) [file pgen.1010950.s013.pdf]

A

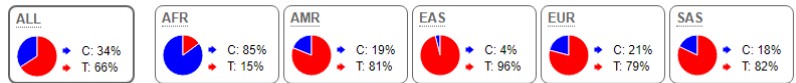

B

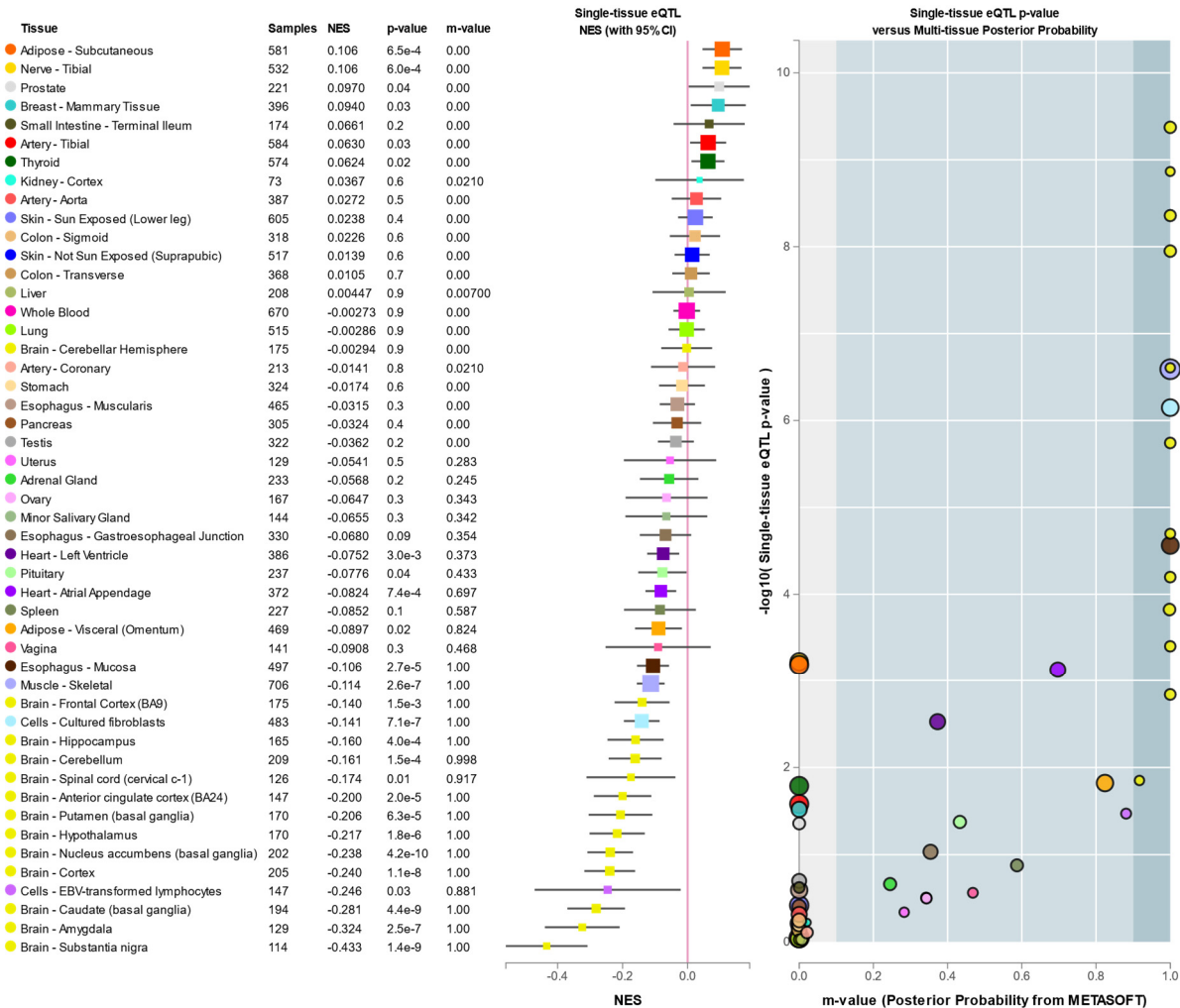

C

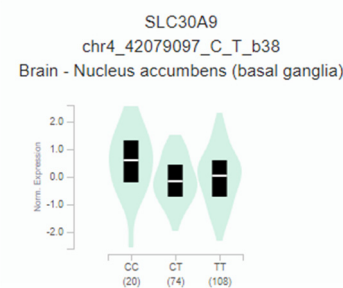

Supplement: S14 Fig — (A) Continental 1000 Genomes Project Phase 3 allele frequencies as retrieved from Ensembl (https://www.ensembl.org/index.html). (B) Multi-tissue eQTL comparison for rs7660223. (C). Differential SLC30A9 expression in the nucleus accumbens according to the rs7660223 genotypes as available at the GTEX portal (https://www.gtexportal.org/home/). NES, normalized effect sizes. (PDF) [file pgen.1010950.s014.pdf]

A

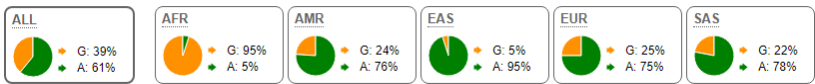

B

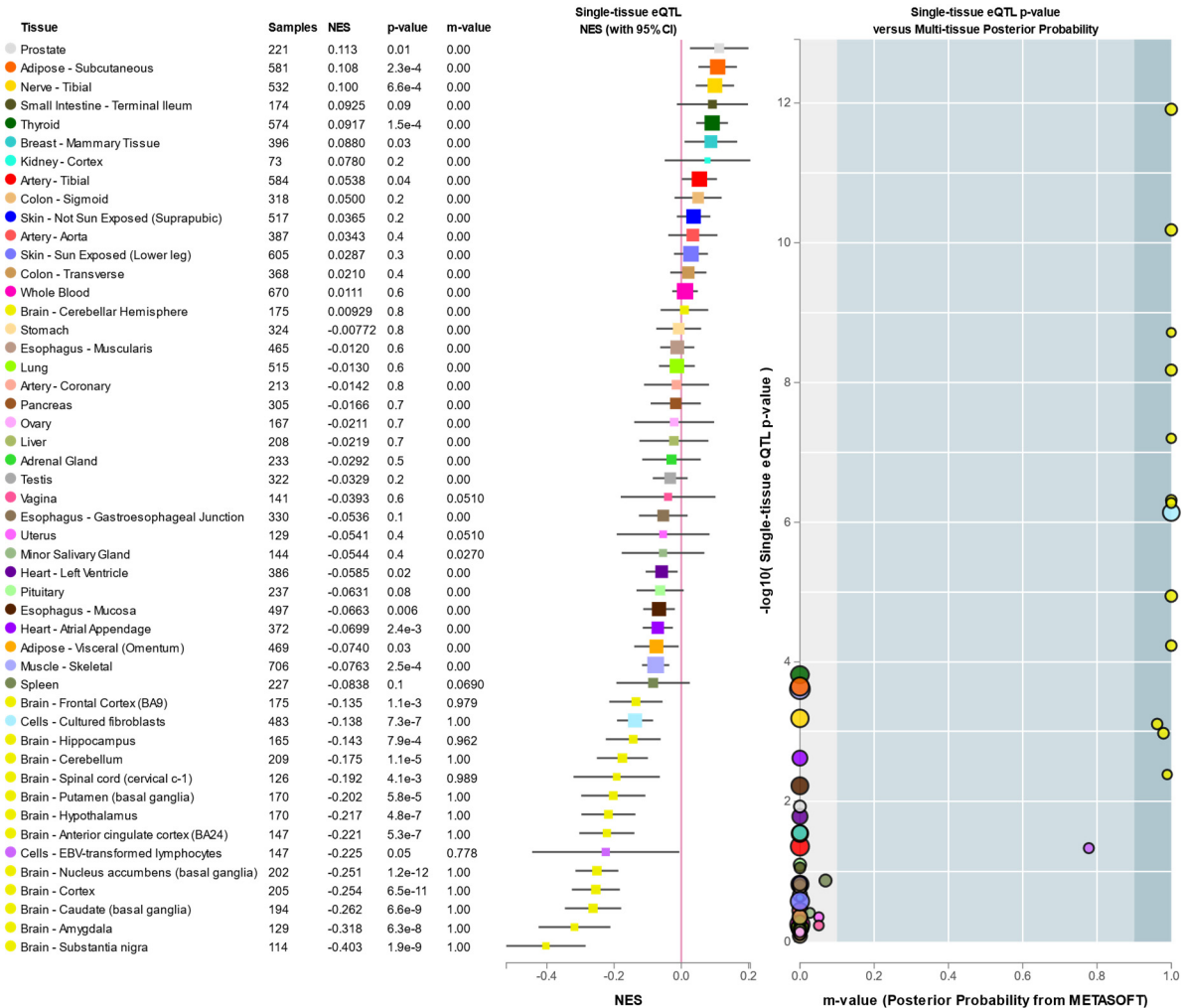

C

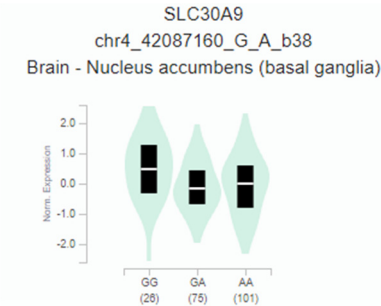

Supplement: S15 Fig — (A) Continental 1000 Genomes Project Phase 3 allele frequencies as retrieved from Ensembl (https://www.ensembl.org/index.html). (B) Multi-tissue eQTL comparison for rs11051. (C). Differential SLC30A9 expression in the nucleus accumbens according to the rs11051 genotypes as available at the GTEX portal (https://www.gtexportal.org/home/). NES, normalized effect sizes. (PDF) [file pgen.1010950.s015.pdf]

A

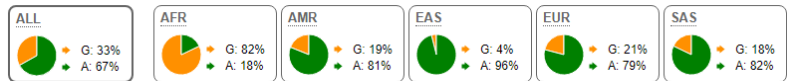

B

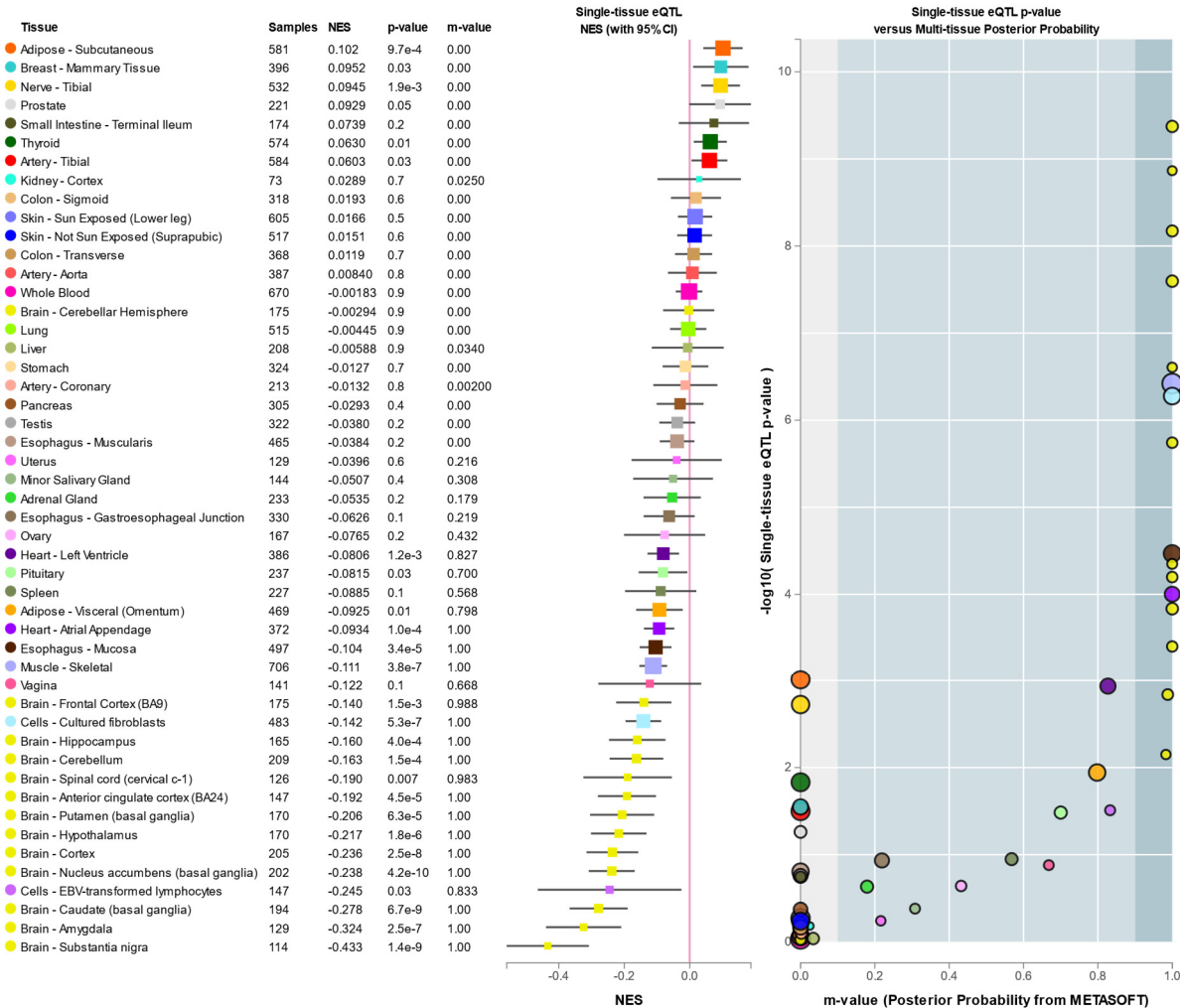

C

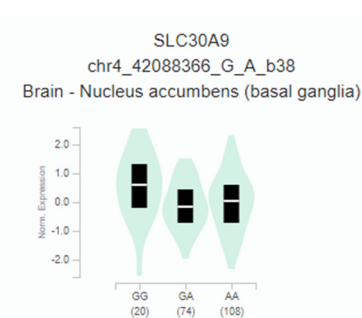

Supplement: S16 Fig — (A) Continental 1000 Genomes Project Phase 3 allele frequencies as retrieved from Ensembl (https://www.ensembl.org/index.html). (B) Multi-tissue eQTL comparison for rs11935648. (C). Differential SLC30A9 expression in the nucleus accumbens according to the rs11935648 genotypes as available at the GTEX portal (https://www.gtexportal.org/home/). NES, normalized effect sizes. (PDF) [file pgen.1010950.s016.pdf]

A

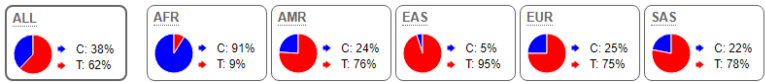

B

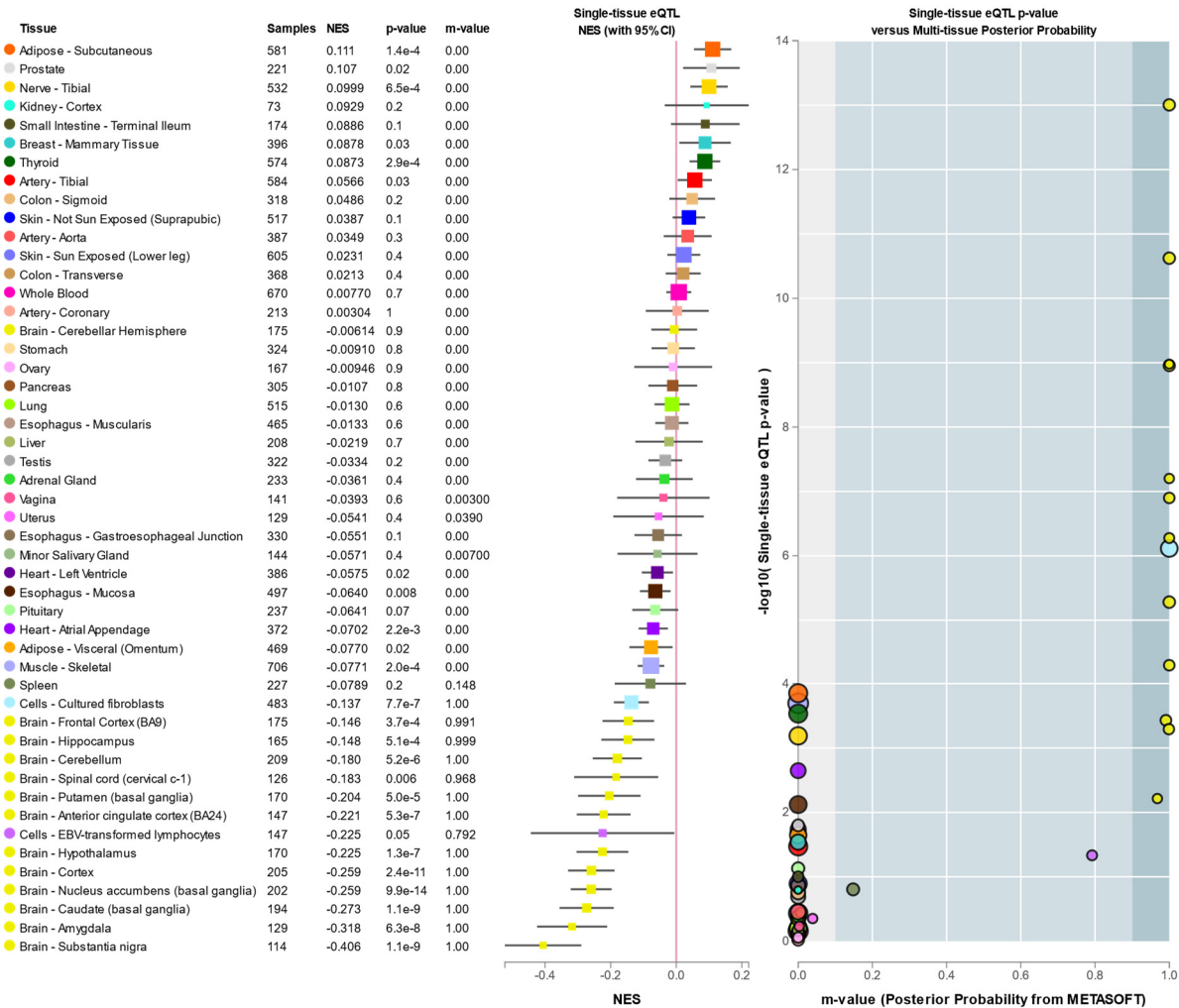

C

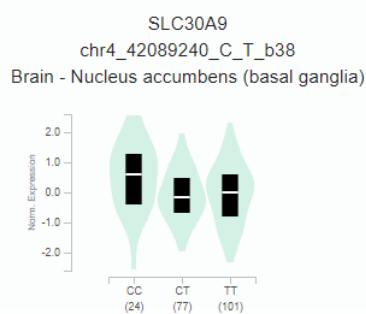

Supplement: S17 Fig — (A) Continental 1000 Genomes Project Phase 3 allele frequencies as retrieved from Ensembl (https://www.ensembl.org/index.html). (B) Multi-tissue eQTL comparison for rs12511999. (C). Differential SLC30A9 expression in the nucleus accumbens according to the rs12511999 genotypes as available at the GTEX portal (https://www.gtexportal.org/home/). NES, normalized effect sizes. (PDF) [file pgen.1010950.s017.pdf]

A

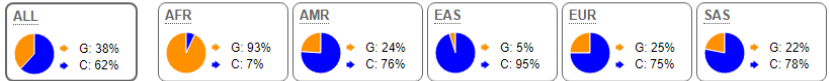

B

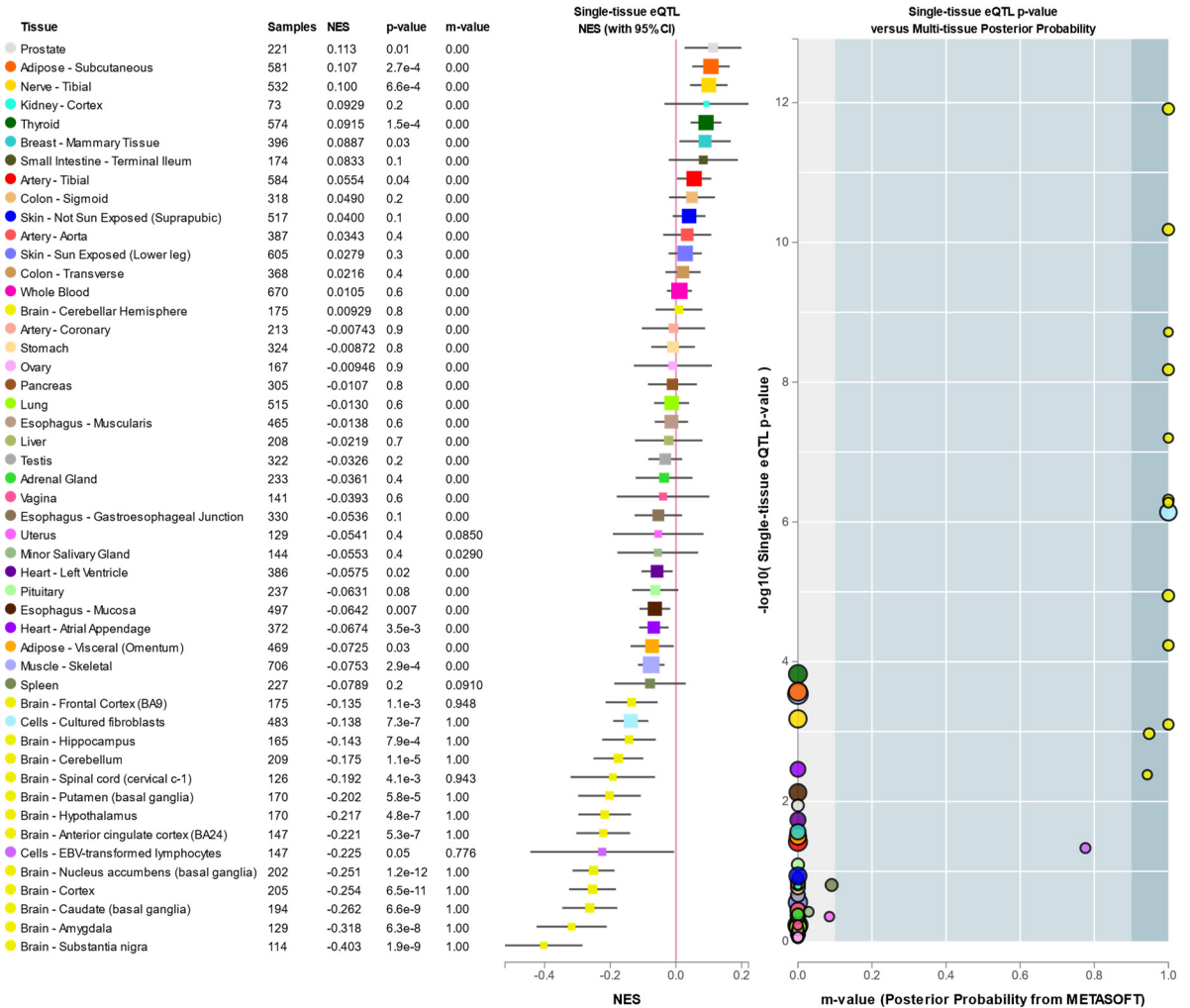

C

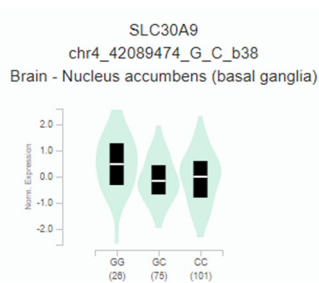

Supplement: S18 Fig — (A) Continental 1000 Genomes Project Phase 3 allele frequencies as retrieved from Ensembl (https://www.ensembl.org/index.html). (B) Multi-tissue eQTL comparison for rs10938178. (C). Differential SLC30A9 expression in the nucleus accumbens according to the rs10938178 genotypes as available at the GTEX portal (https://www.gtexportal.org/home/). NES, normalized effect sizes. (PDF) [file pgen.1010950.s018.pdf]

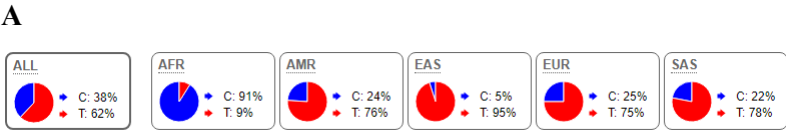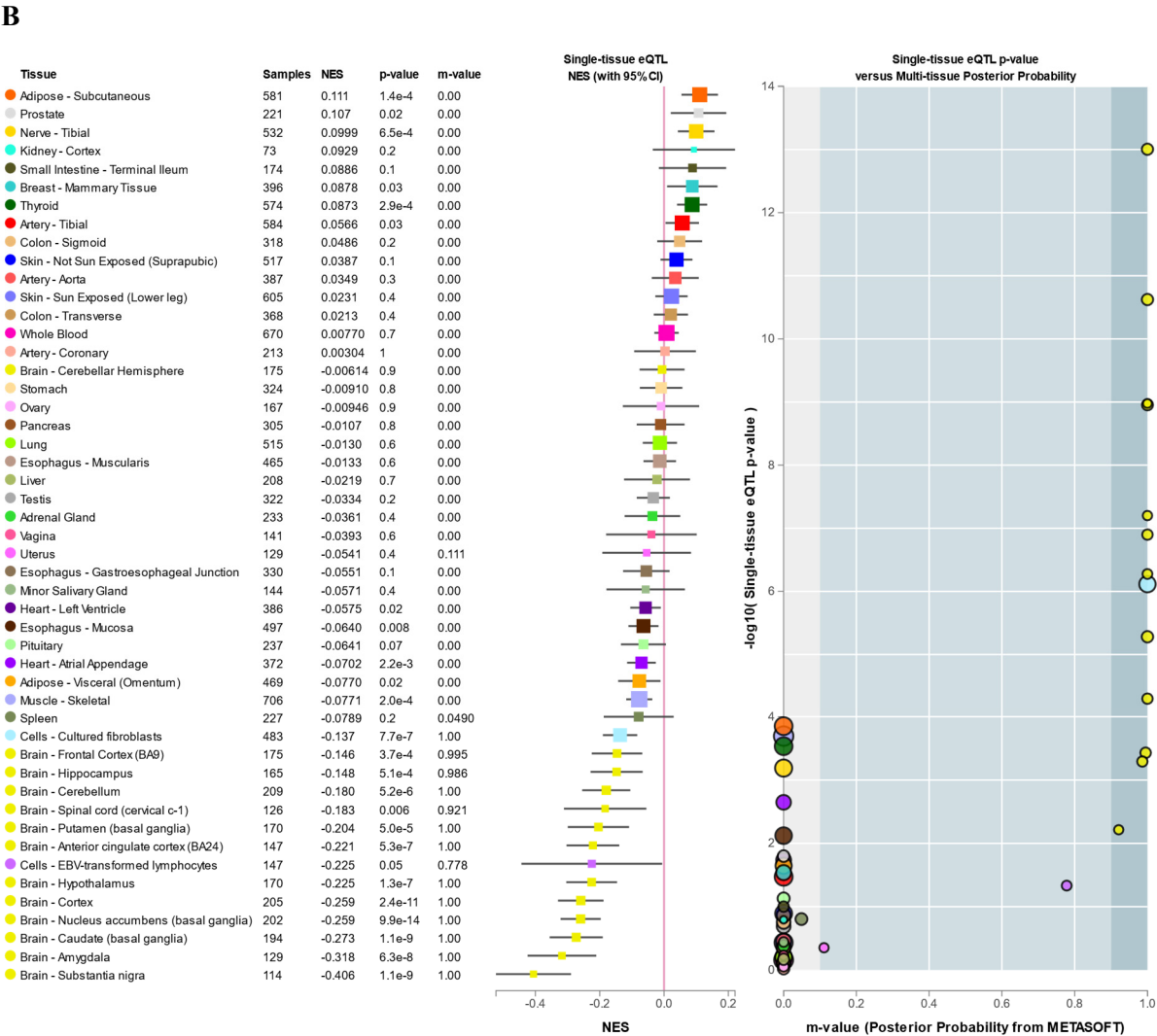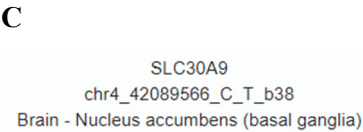

Supplement: S19 Fig — (A) Continental 1000 Genomes Project Phase 3 allele frequencies as retrieved from Ensembl (https://www.ensembl.org/index.html). (B) Multi-tissue eQTL comparison for rs12512101. (C). Differential SLC30A9 expression in the nucleus accumbens according to the rs12512101 genotypes as available at the GTEX portal (https://www.gtexportal.org/home/). NES, normalized effect sizes. (PDF) [file pgen.1010950.s019.pdf]

A

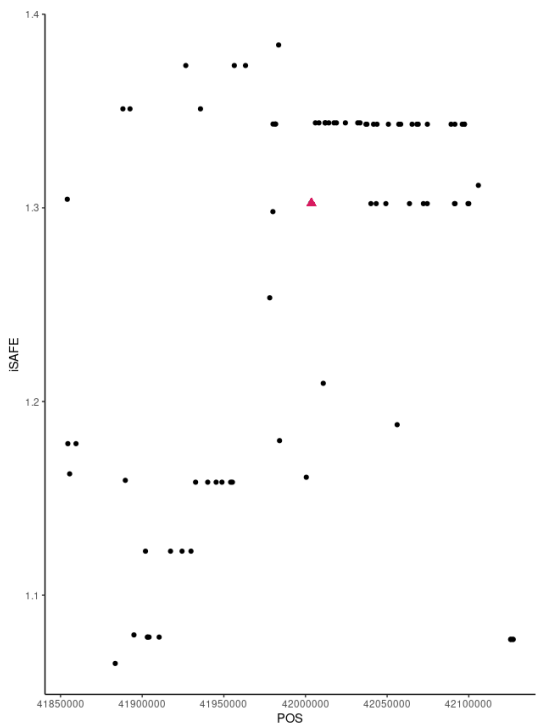

# B

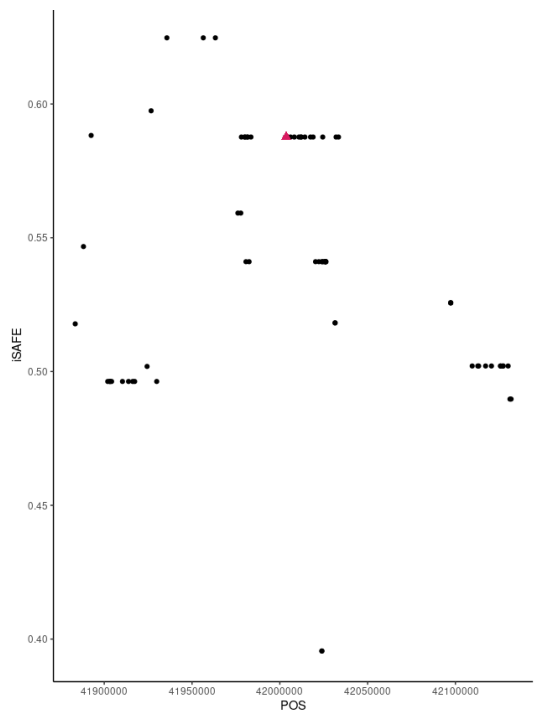

Supplement: S20 Fig — iSAFE values were computed within 300 kb (chr4: 41853671–42153671, GRCh37/hg19) centered on rs1047626, shown in red. A. iSAFE values in CHB. B. iSAFE values in CEU. Further details on the SNP annotation and iSAFE values are available in S2 and S3 Tables. (PDF) [file pgen.1010950.s020.pdf]

**A**

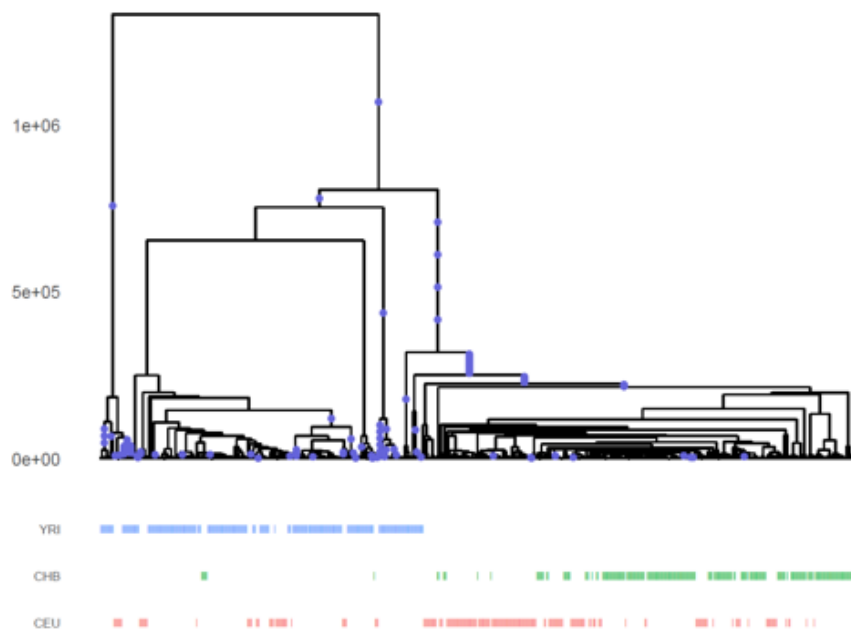

**B**

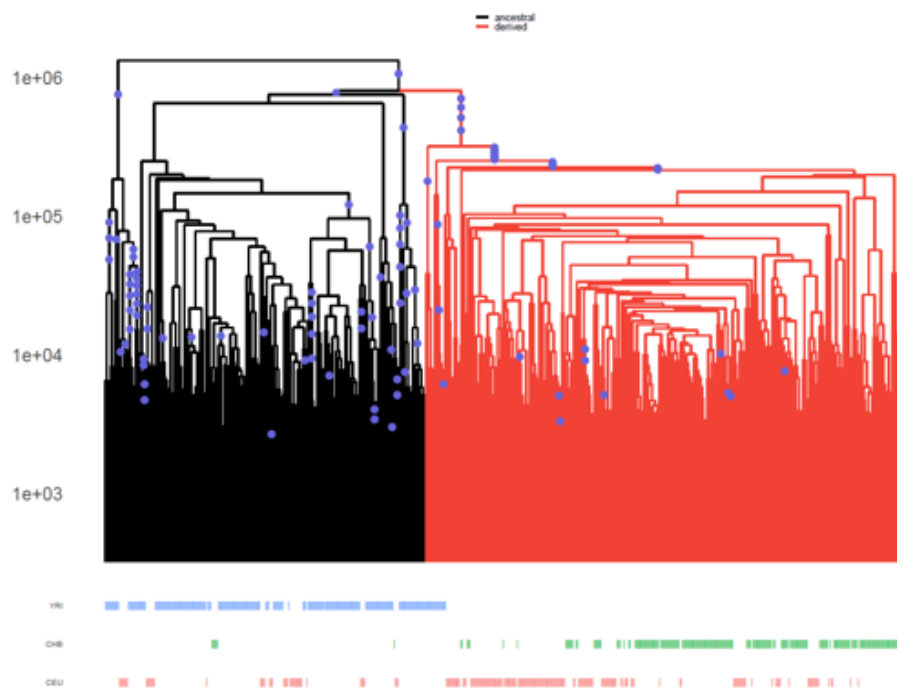

Supplement: S21 Fig — A) Marginal tree corresponding to the rs1047626 flanking region (chr4: 42002370–42004739; GRCh37/hg19) at SLC30A9. The derived allele at this SNP expanded rapidly in CHB and CEU, which is indicative of positive selection. B) Tree of interest highlighting those lineages carrying the derived allele at rs1047626. (PDF) [file pgen.1010950.s021.pdf]

**CEU:**  $s = 0.00171$ ;  $\log LR = 10.93$

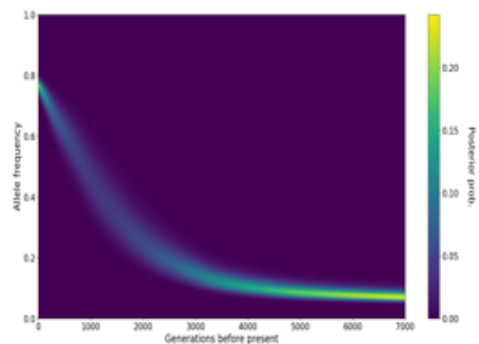

**CHB:**  $s = 0.011343$ ;  $\log LR = 5.68$

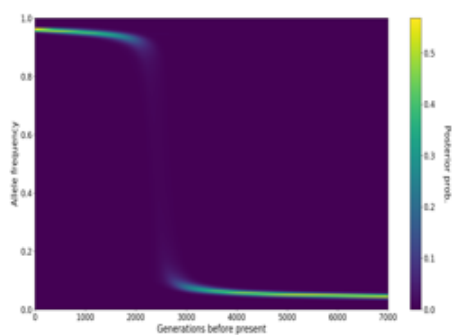

**FIN:**  $s = 0.00210$ ;  $\log LR = 9.56$

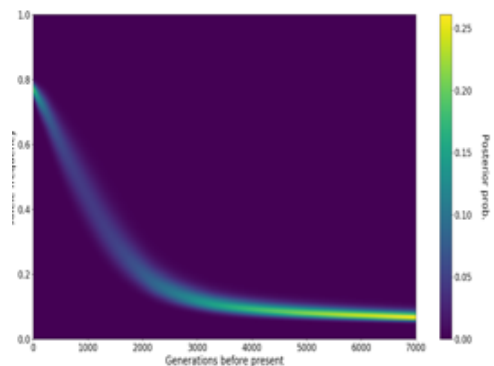

**PEL:**  $s = 0.00222$ ;  $\log LR = 10.69$

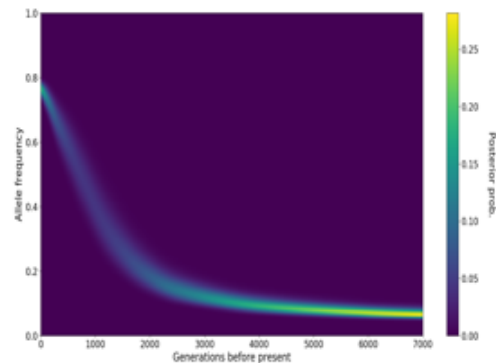

**JPT:**  $s = 0.00356$ ;  $\log LR = 13.03$

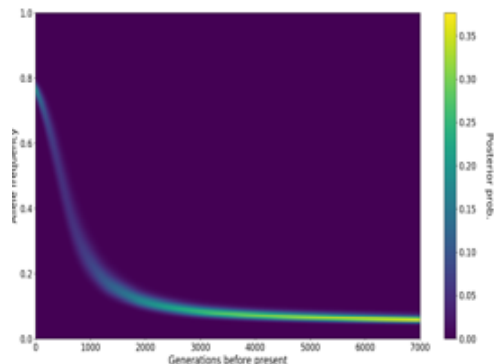

**PJL:**  $s = 0.00229$ ;  $\log LR = 13.9286$

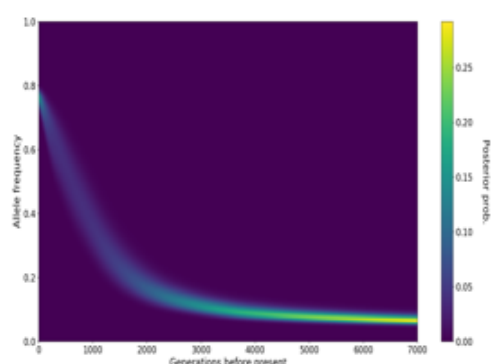

Supplement: S22 Fig — CEU, Utah residents (CEPH) with Northern and Western European ancestry; CHB, Han Chinese in Beijing, China; FIN, Finnish in Finland; PEL, Peruvian in Lima, Peru; JPT, Japanese in Tokyo, Japan; PJL, Punjabi in Lahore, Pakistan. (PDF) [file pgen.1010950.s022.pdf]

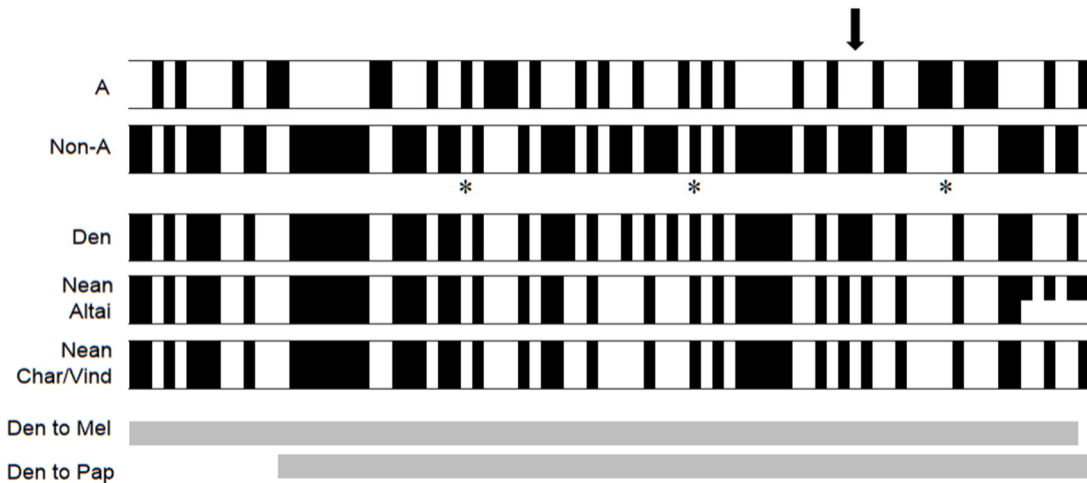

Supplement: S23 Fig — Schematic representation of the two major human haplotypes as defined by 84 SNPs in high linkage disequilibrium (r2>0.8) with rs1047626 in CEU and CHB along the putatively inferred introgressed region (chr4: 41,977,828–42,048,441; GRCh38) and the corresponding allele states found in four high coverage Denisovan and Neanderthal genomes. Derived states are indicated in black, and ancestral alleles in white. Den to Papuan and Den to Melanesian indicate Denisovan introgression segments previously described in Melanesians [34] and Papua New Guineans [35]. The black arrow point to the rs1047626 position. GWAS hits are indicated with an asterisk (see S4 Table for details). (PDF) [file pgen.1010950.s023.pdf]

A

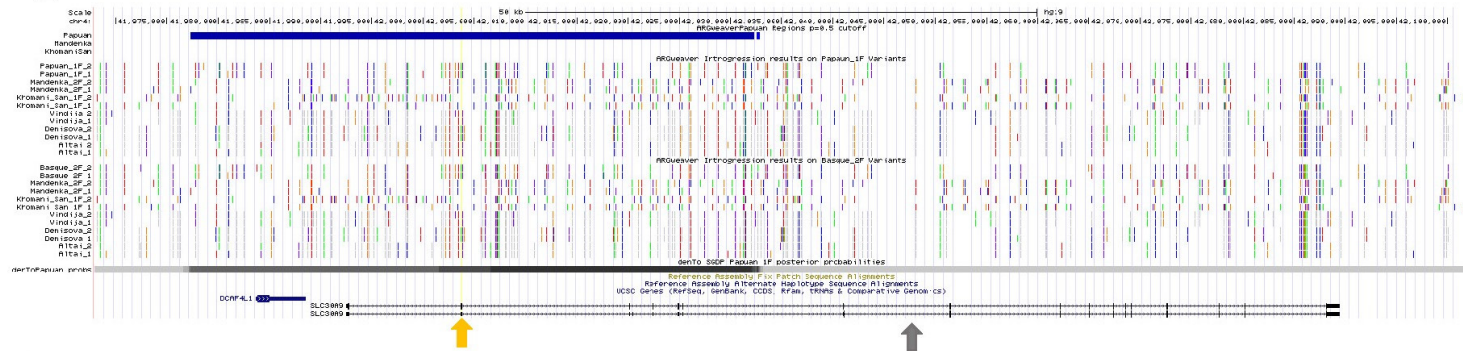

B

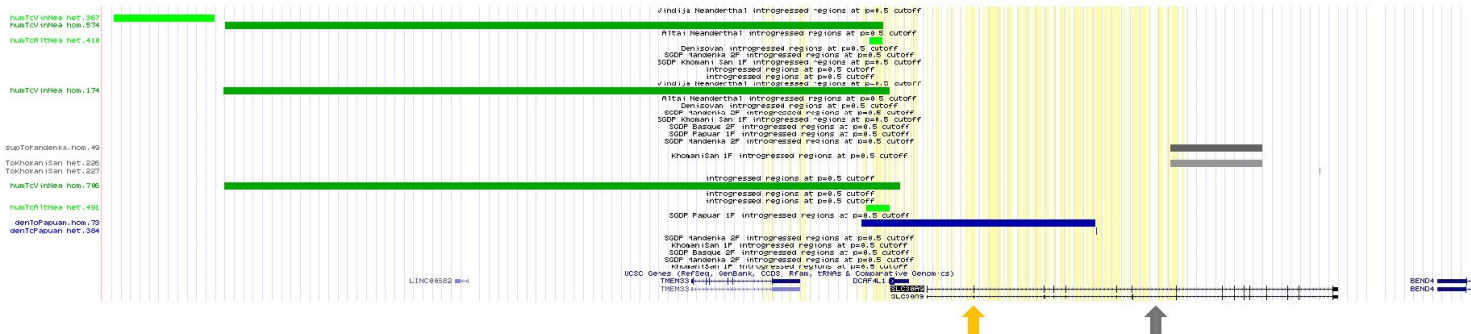

Supplement: S24 Fig — A) ARGweaver Denisovan introgression results track in modern Papuans as available at the UCSC Genome Browser on Human GRCh37/hg19 and according to the methods and model described in Hubisz et al. (2020) [35]. Arrows in yellow and grey indicate the positions of rs1047626 and rs4861157, respectively. The “Region” track shows the predicted Denisovan introgressed regions in blue using a posterior probability cut-off of 0.5. For each individual, variants used in the analysis are shown above with alternating colours indicating variant alleles. When chimpanzee alignments are available, the non-chimp allele is coloured; otherwise, the minor allele is coloured. B) Overview of introgression tracks surrounding the SLC30A9 gene region as inferred by ARGweaver and available at the UCSC Genome Browser. In blue, Denisovan to Papuan introgression at p = 0.5 cut-off (chr4: 41977301–42032370); in green, human to Vindija/Altaic Neanderthal introgression at p = 0.5 cut-off (chr4: 41827151–41986310, chr4: 41801111–41824760 and chr4: 41978271–41983950); in dark grey, Super-Archaic hominin to Mandenka 2F introgression regions at p = 0.5 cutoff (chr4:42050001–42071760); in light grey, Super-Archaic hominin to KhomaniSan 1F introgressed regions at p = 0.5 cut-off (chr4:42050001–42071760). (PDF) [file pgen.1010950.s024.pdf]

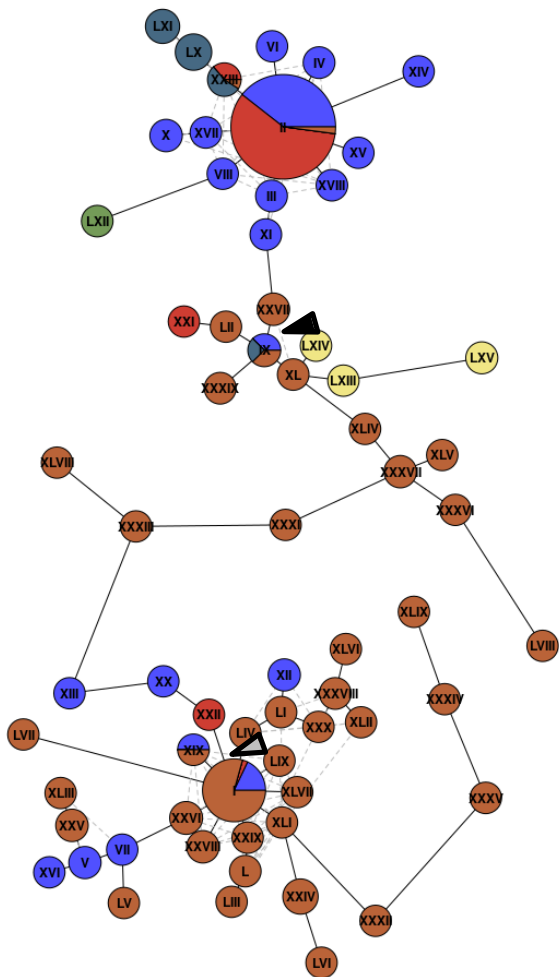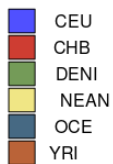

Supplement: S25 Fig — Haplotypes were defined by the complete set of SNPs available with a homozygous genotype in the Denisovan genome along the putatively introgressed region (chr4: 41,977,828–42,048,441; GRCh38). Pie charts show the frequency of each haplotype among YRI, CEU, CHB, and Oceanians. Denisovan and Neanderthal haplotypes are show in green and yellow, respectively. Arrow in black, position of the rs1047626 substitution; arrow in grey, position of the rs4861157 polymorphism. Haplotype frequencies, and polymorphic positions defining each haplotype are available in S5 Table. For nucleotide pairwise distances between haplotypes, see S6 Table. (PDF) [file pgen.1010950.s025.pdf]

A

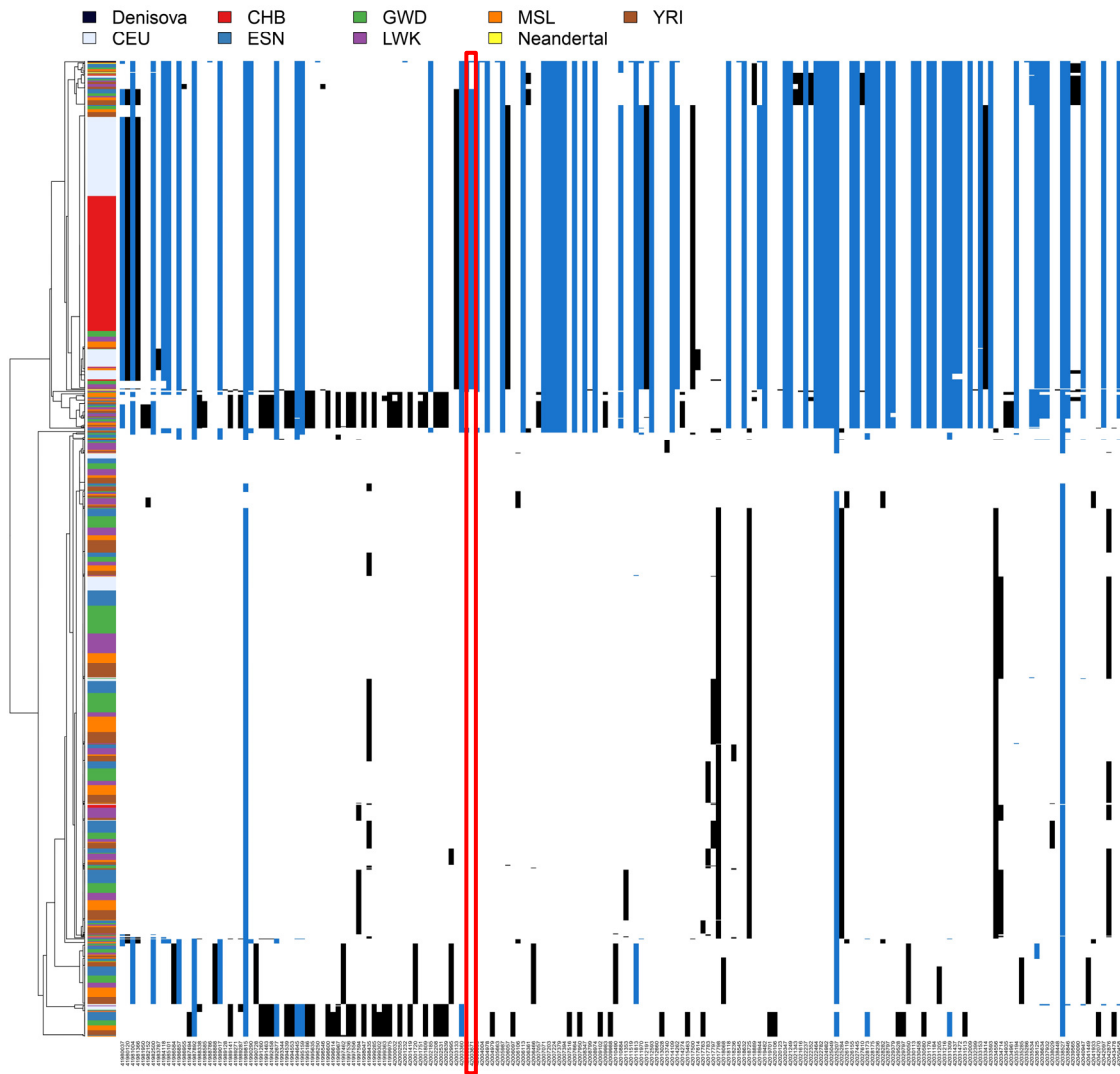

B

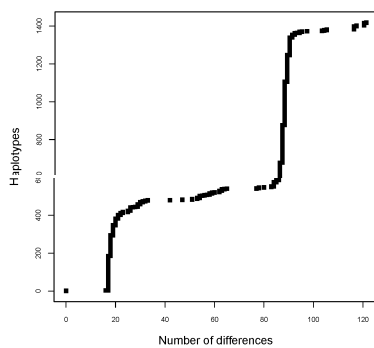

Supplement: S26 Fig — Note that Oceanians and Chagyrskaya were not included in the analysis to maximize the number of SNPs in the haplotypes. A total of 195 SNPs along the SLC30A9 region (chr4: 41975811–42046424; GRCh37) defined 97 unique haplotypes when considering the three remaining high coverage archaic genomes (Altai, Vindija and Denisova), and the CEU and CHB populations together with an extended panel of African populations (ESN, Esan in Nigeria; YRI, Yoruba in Ibadan, Nigeria; MSL, Mende in Sierra Leone; GWD, Gambian Mandinka; LWK, Luhya in Webuye, Kenya). Note that all sites with a maximum within-population minor allele frequency below 0.05 were removed. A) Haplotypes depicted in decreasing order according to their similarity to the Denisovan genome, shown in the top. Each column corresponds to a SNP. The white colour in each cell indicates the ancestral allele; blue, indicates a derived allele shared with the Denisovan genome; whereas black indicates a derived allele that is not shared with the Denisova. The red box highlights the location of rs1047626. B) Number of differences observed to the Denisovan haplotype (see values and individual haplotype codes per population in S8 Table). (PDF) [file pgen.1010950.s026.pdf]

A

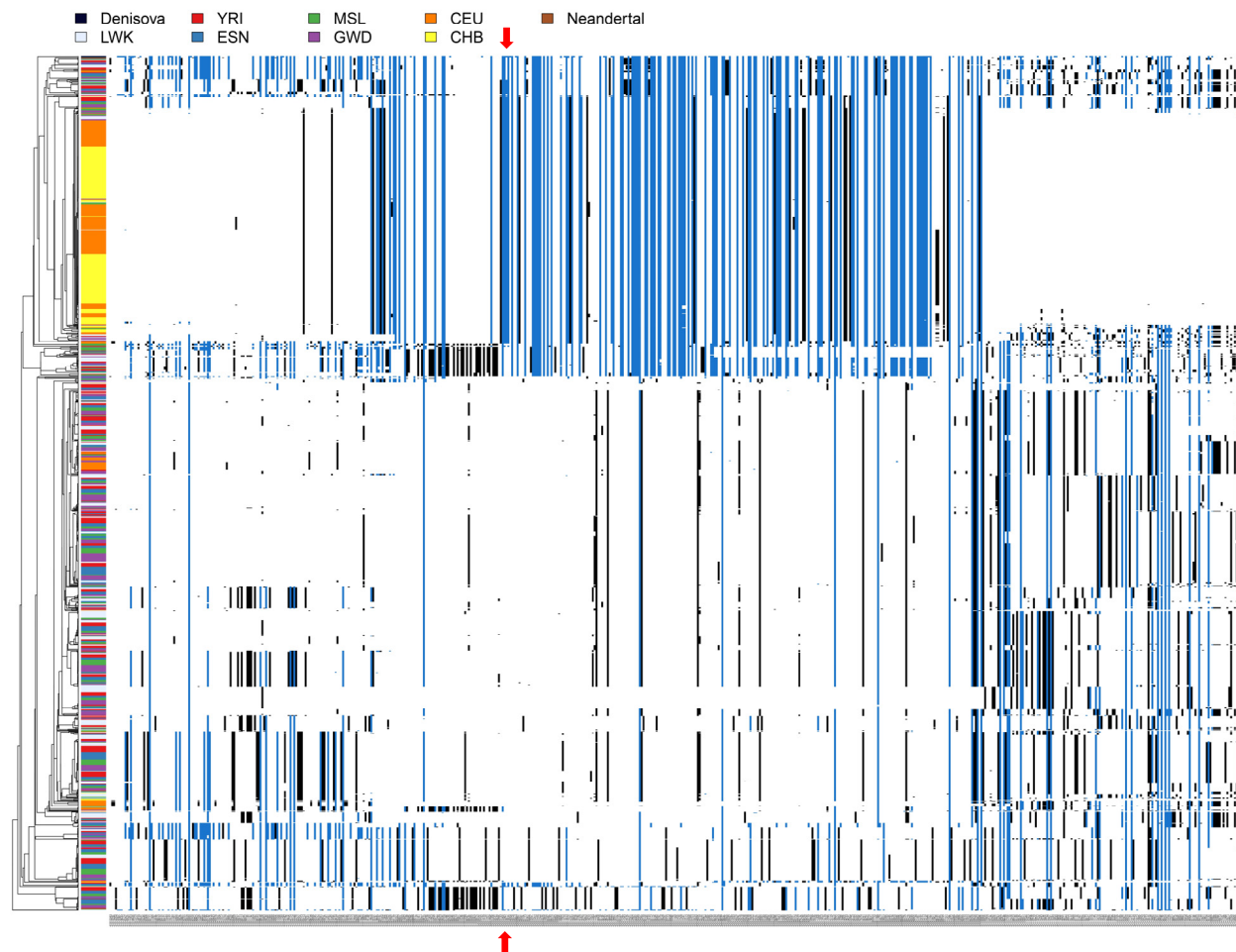

B.

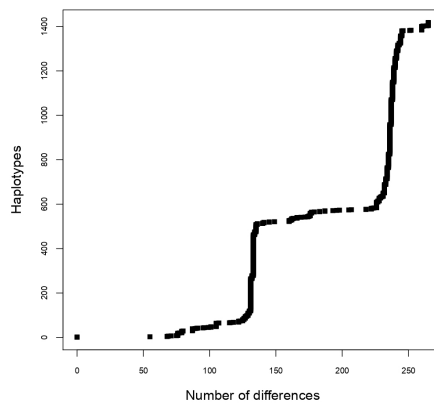

Supplement: S27 Fig — Note that Oceanians and Chagyrskaya were not included in the analysis to maximize the number of SNPs in the haplotypes. A total of 603 SNPs in an extended genomic region around the SLC30A9 gene (chr4:41905811–42146424; GRCh37) defined 347 unique haplotypes when considering the three remaining high coverage archaic genomes (Altai, Vindija and Denisova), and the CEU and CHB populations together with an extended panel of African populations (ESN, Esan in Nigeria; YRI, Yoruba in Ibadan, Nigeria; MSL, Mende in Sierra Leone; GWD, Gambian Mandinka; LWK, Luhya in Webuye, Kenya). Note that all sites with a maximum within-population minor allele frequency below 0.05 were removed. A) Haplotypes depicted in decreasing order according to their similarity to the Denisovan genome, shown in the top. Each column corresponds to a SNP. The white colour in each cell indicates the ancestral allele; blue, indicates a derived allele shared with the Denisovan; whereas black indicates a derived allele that is not shared with the Denisova. The red arrow highlights the location of rs1047626. B) Number of differences observed to the Denisovan haplotype (see values and individual haplotype codes per population in S9 Table). (PDF) [file pgen.1010950.s027.pdf]

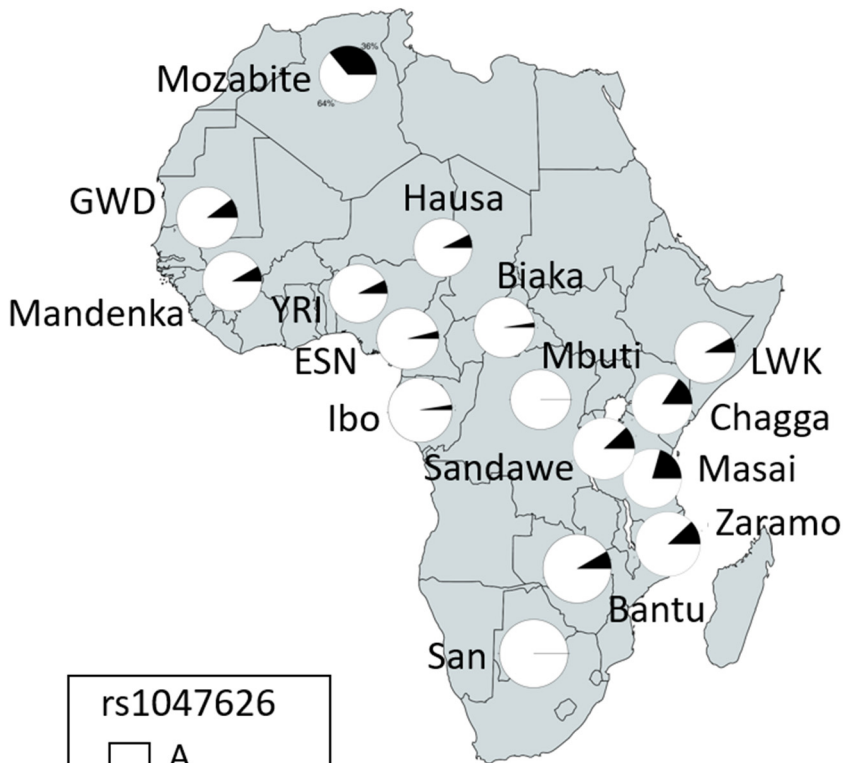

rs1047626

□ A

■ G

Supplement: S28 Fig — Allele frequencies for the Mbuti, Mozabite, Hausa, San, Ibo, Chagga, Masai, Sandawe and Zaramo were compiled from the ALFRED database (https://alfred.med.yale.edu/alfred/index.asp) [74], for ESN, GWD, LWK, MSL and YRI from the 1000 Genomes Project [63] data available at Ensembl (https://www.ensembl.org/index.html), and for Biaka, Bantu, and Mandenka from the Human Genome Diversity Panel (HGDP-CEPH) [37]. (PDF) [file pgen.1010950.s028.pdf]

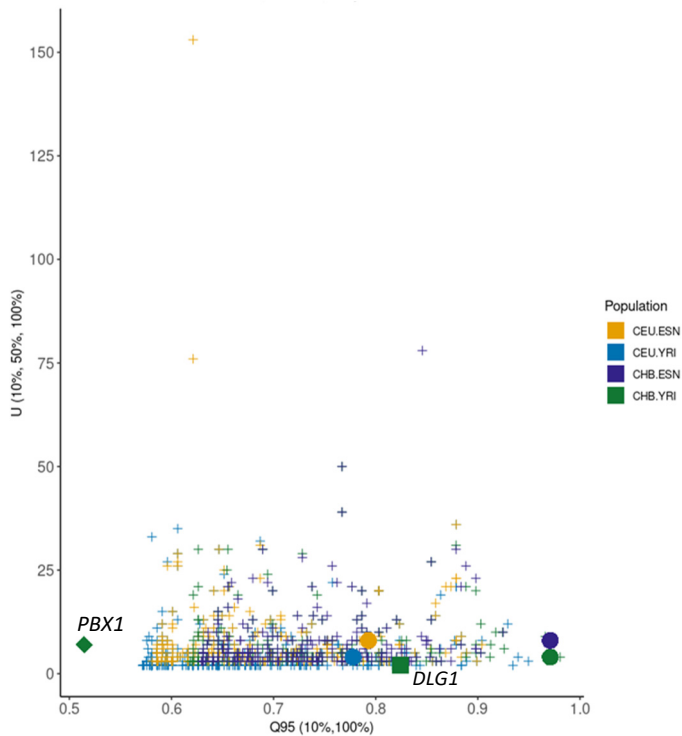

Supplement: S29 Fig — Circles denote the U (10%, 50%, 100%) and Q95 (10%, 100%) values in the SLC30A9 region, whereas the square and the rombe denote the statistics values in two candidate regions for Denisovan introgression in CHB [41]. (PDF) [file pgen.1010950.s029.pdf]

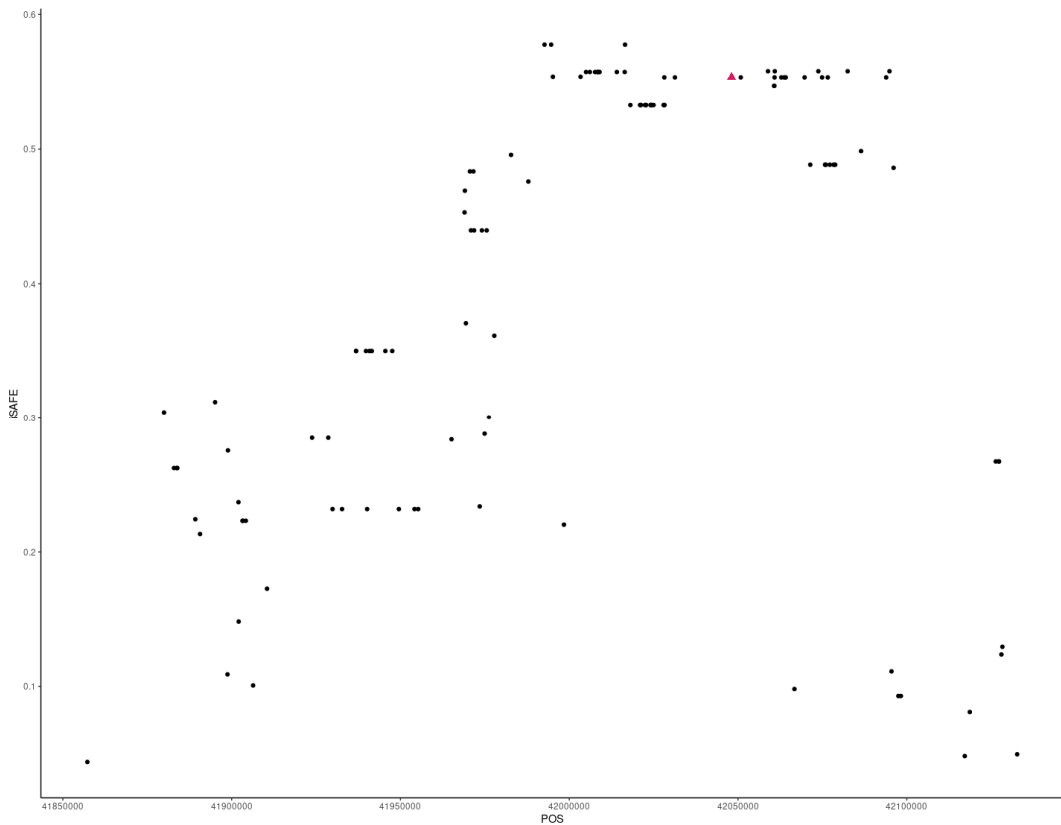

Supplement: S30 Fig — iSAFE values computed along the SLC30A9 candidate region for selection (chr4: 41853671–42153671, GRCh37/hg19) in the Yoruba population. In red, iSAFE value for rs4861157. Complete details on the SNP annotation and iSAFE values are available in S15 Table. (PDF) [file pgen.1010950.s030.pdf]

**A**

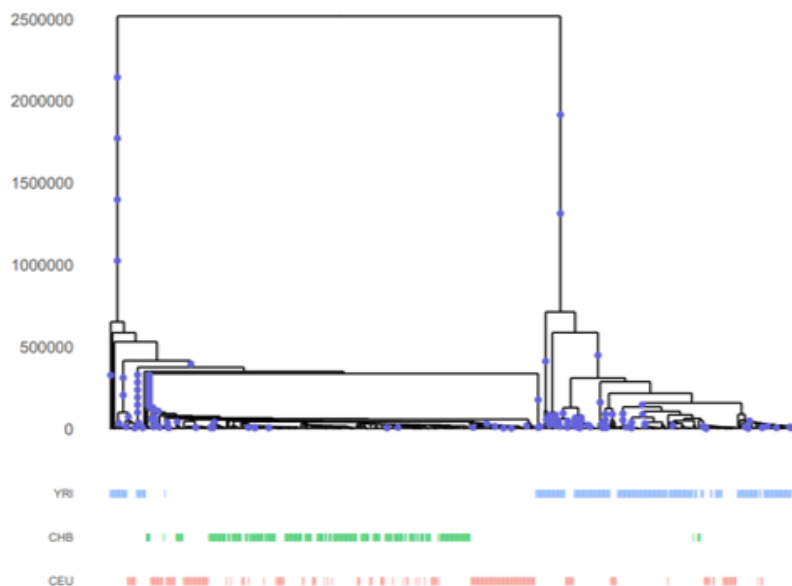

**B**

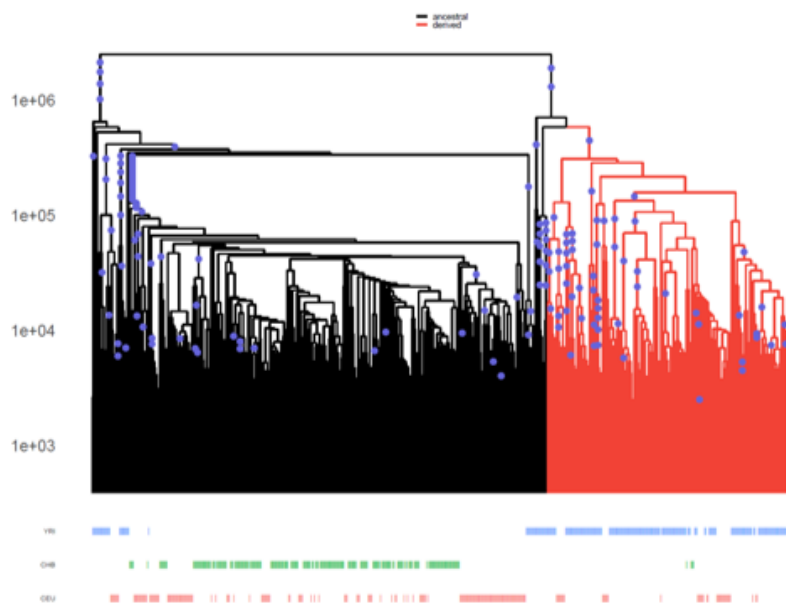

Supplement: S31 Fig — A) Marginal tree corresponding to the rs4861157 flanking region (chr4: 42022464–42025285; GRCh37/hg19) at SLC30A9. The derived allele at this SNP expanded rapidly in YRI, which is indicative of positive selection. B) Tree of interest highlighting lineages carrying the derived allele at rs4861157. (PDF) [file pgen.1010950.s031.pdf]

A

## GTEx IGV Browser

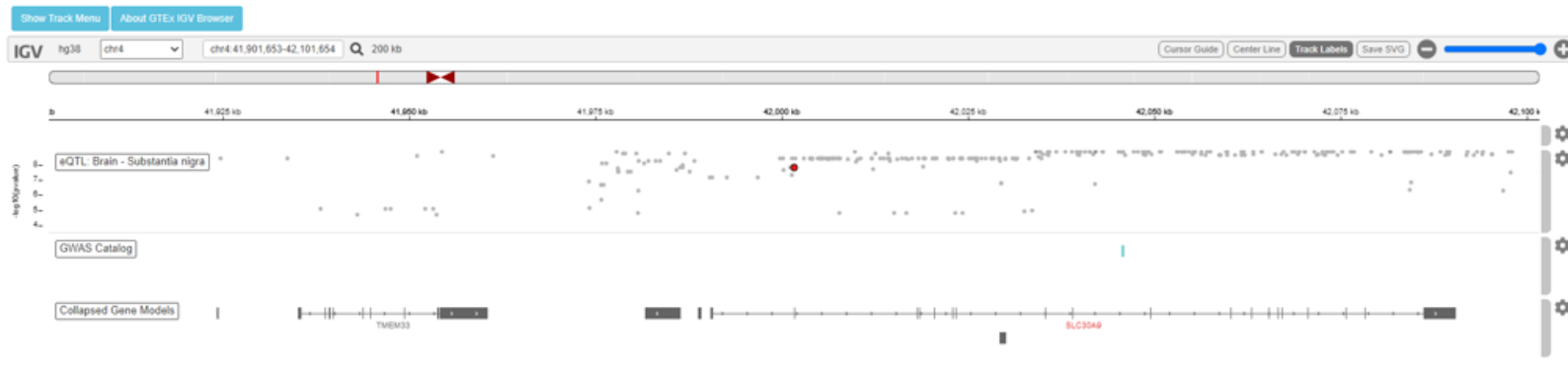

B

## GTEx IGV Browser

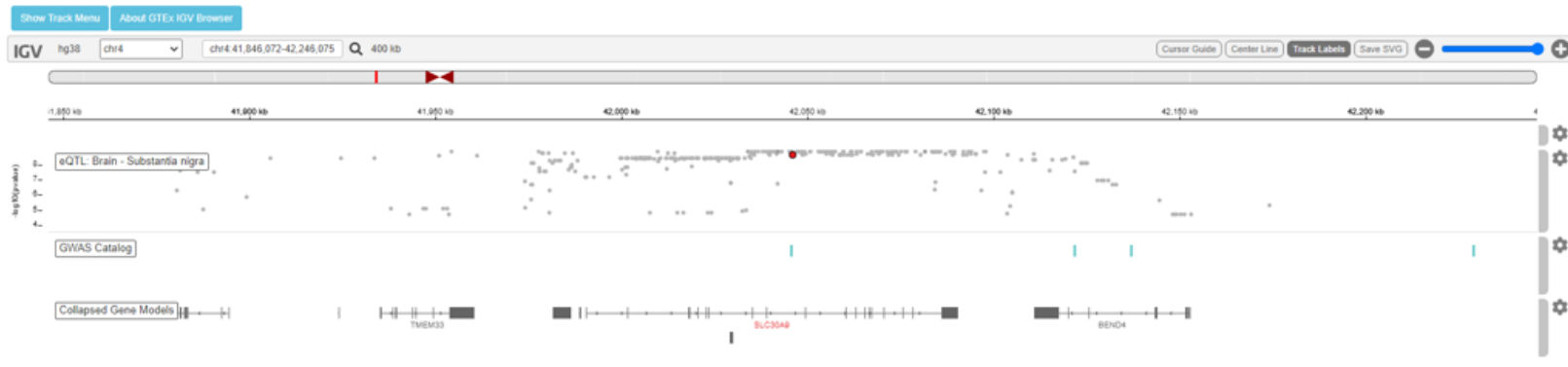

Supplement: S32 Fig — A) GTEx IGV Browser image showing rs1047626 significance as an eQTL (red dot). B) GTEx IGV Browser image showing rs4861157 significance as an eQTL (red dot). (PDF) [file pgen.1010950.s032.pdf]

A

1000 Genomes Project Phase 3 allele frequencies

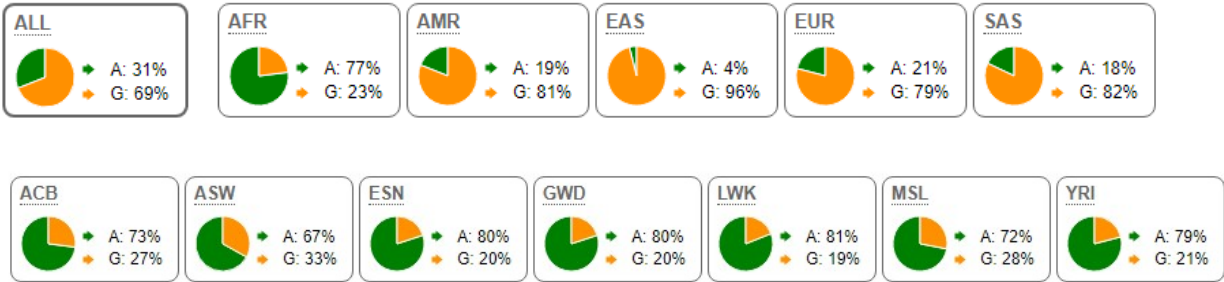

B

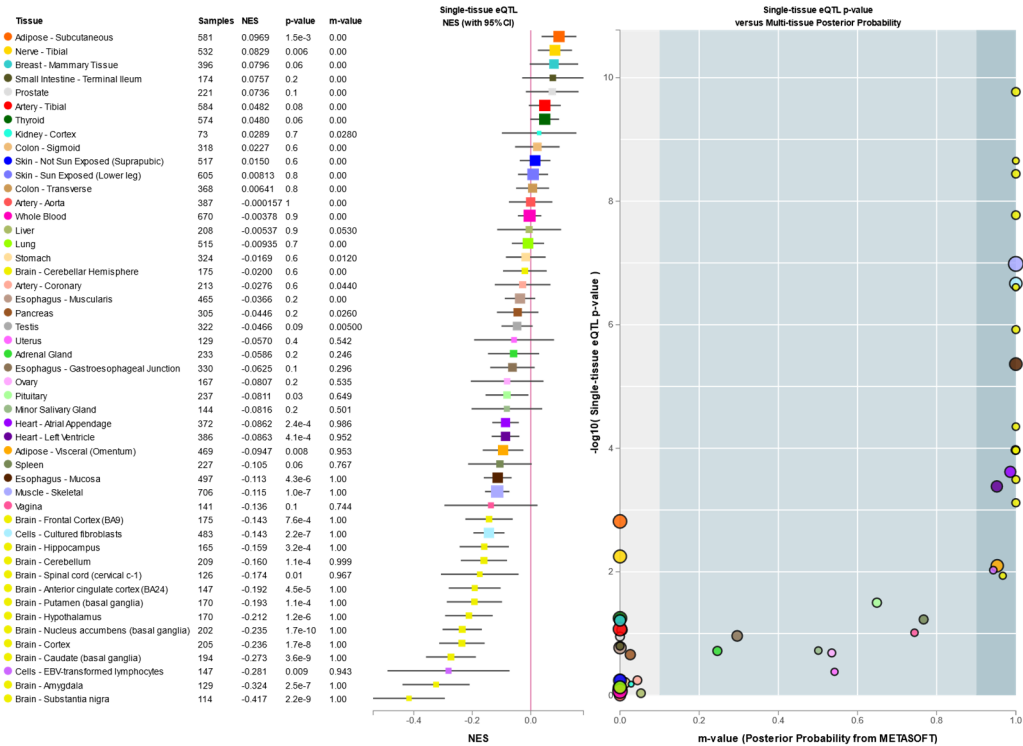

Supplement: S33 Fig — (A) Continental and African sub-populations 1000 Genomes Project Phase 3 allele frequencies as retrieved from Ensembl (https://www.ensembl.org/index.html). (B) Multi-tissue eQTL comparison for rs4861157. (PDF) [file pgen.1010950.s033.pdf]

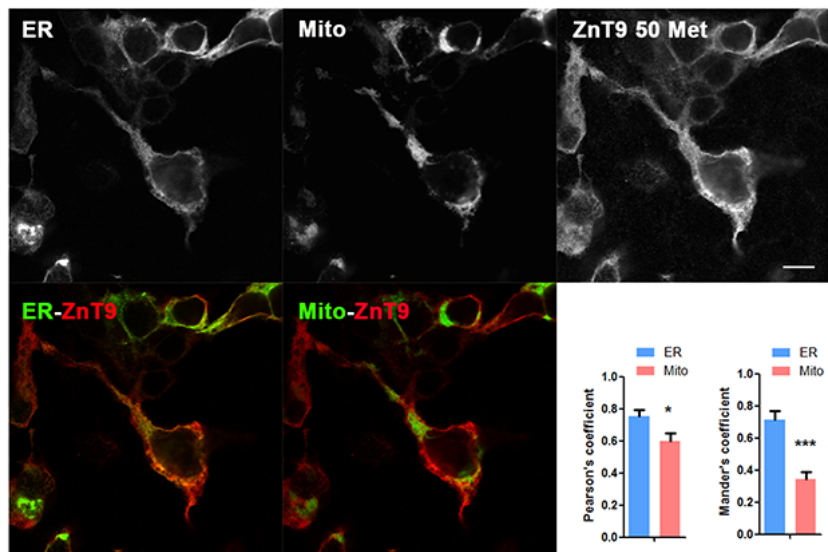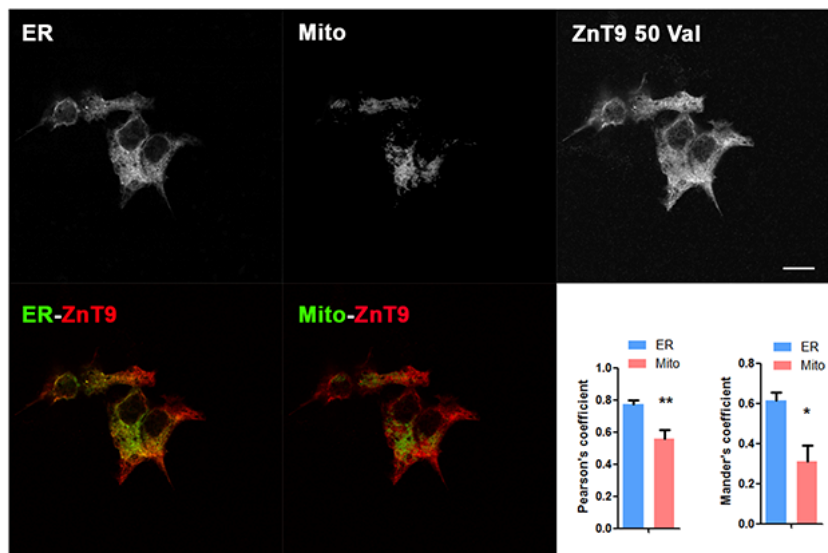

Supplement: S34 Fig — Representative pictures of colocalization of ZnT9-50Met (A) and ZnT9-50Val (B) (both in red) with a double fluorescent reporter plasmid from the endoplasmic reticulum and the mitochondria (both in green in separate images). Scale bar = 10 μm. Representation of Pearson’s correlation coefficient and Mander’s overlap coefficient for quantifying the colocalization of ZnT9-50Met (n = 5) and ZnT9-50Val (n = 4) with endoplasmic and mitochondria. *** p < 0.001, ** p < 0.01, * p < 0.05. See further statistical details in S1 Data. (PDF) [file pgen.1010950.s034.pdf]

**A**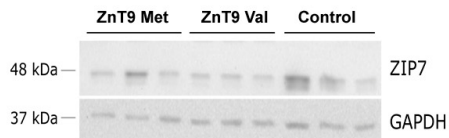**B**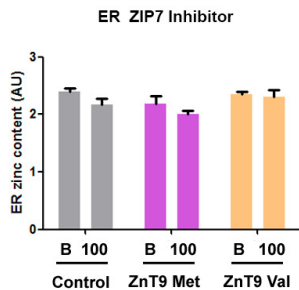**C**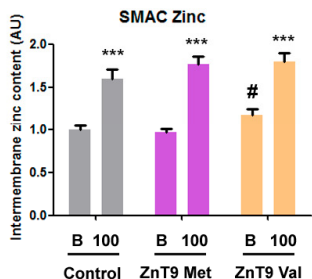**D**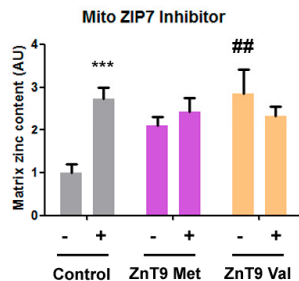**E**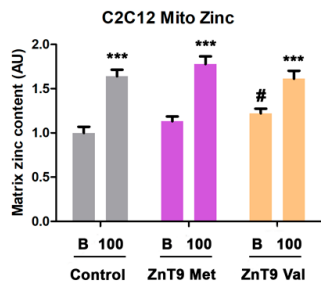**F**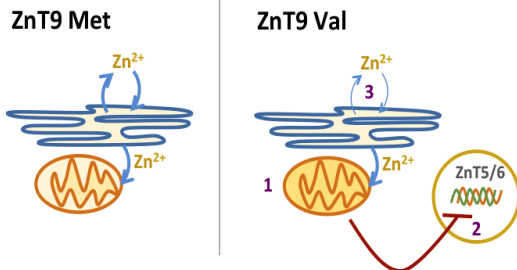

Supplement: S35 Fig — (A) Representative Western blot against ZIP7 and GAPDH in HEK293 cells transfected with ZnT9-50Met, ZnT9-50Val, or an empty vector. (B) Evaluation of endoplasmic zinc content in HEK293 cells using an ER fluorescent zinc sensor (ER-ZapCY1) in basal and 100 μM ZnSO4 in the presence of DMSO and Zip7 blocker (n = 12–19). (C) Evaluation of zinc mitochondrial intermembrane space content in HEK293 cells using SMAC-Gn2Zn probe in conditions of 10 μM and 100 μM ZnSO4 (n = 8–9); *** p<0.001 using t-test between basal and 100 μM zinc conditions, # p<0.05 using Bonferroni-corrected ANOVA between transfection conditions. (D) Evaluation of zinc mitochondrial matrix content in HEK293 cells using Mito-cCherry-Gn2Zn incubating 40 min in the presence of DMSO or the Zip7 blocker (n = 6–8). *** p<0.001 using t-test between DMSO and blocker, ## p<0.05 using Bonferroni-corrected ANOVA between transfection conditions. (E) Evaluation of zinc mitochondrial matrix content in C2C12 cells using Mito-cCherry-Gn2Zn incubating 40 min with basal and 100 μM ZnSO4 conditions (n = 21–28); *** p<0.01 using t-test between basal and 100 μM zinc conditions, # p<0.05 using Bonferroni-corrected ANOVA between transfection conditions. (F) Schematic model of the effect of the ZnT9 variants in zinc homeostasis. 1, ZnT9 Val expressing cells contain higher mitochondrial zinc content. Mitochondrial zinc is dependent of endoplasmic reticulum zinc content. 2, A higher mitochondrial zinc could be compensated in order to avoid overload with the transcriptional repression of the endoplasmic zinc importers ZnT5 and ZnT6. 3, ER zinc content is not altered because the zinc exporter Zip7 is the master regulator of ER zinc content. See further statistical details in S1 Data. (PDF) [file pgen.1010950.s035.pdf]

**A**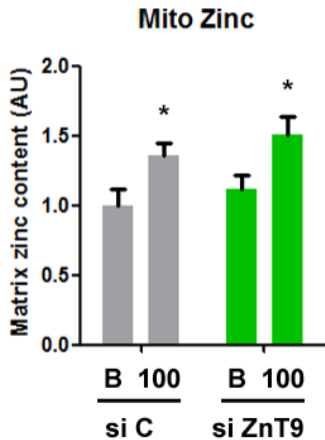**B**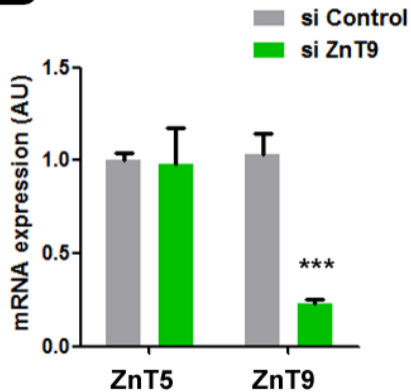

Supplement: S36 Fig — (A) Evaluation of zinc mitochondrial matrix content in siRNAControl (siC) and siRNA ZnT9 (siZnT9) transfected cells using Mito-cCherry-Gn2Zn 40 min with basal and 100 μM ZnSO4 conditions (n = 10–11); * p<0.05 using t-test. (B) RNA expression analysis of ZnT5 and ZnT9 cells transfected with siRNAControl (siC) and siRNA ZnT9 (siZnT9). 2-(DDCT) plotted using GAPDH as the housekeeping gene (n = 6); *** p<0.001 using t-test. See further statistical details in S1 Data. (PDF) [file pgen.1010950.s036.pdf]

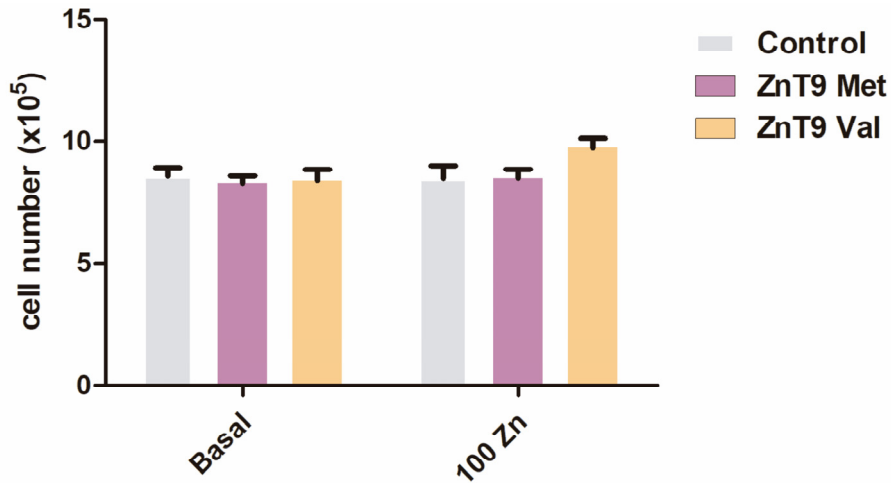

Supplement: S37 Fig — Cell counting assay in cells transfected with ZnT9-50Met, ZnT9-50Val, or an empty vector incubated for 24h at basal and 100 μM ZnSO4 conditions. Data are reported as mean ± SE (error bars) (n = 8–12). No statistically significant differences were found. See further statistical details in S1 Data. (PDF) [file pgen.1010950.s037.pdf]

**A**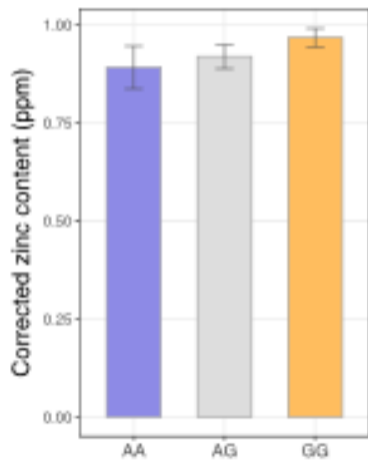**B**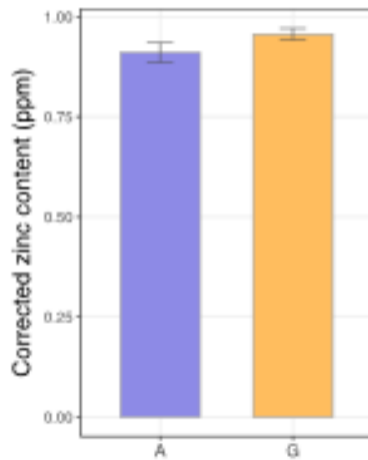

Supplement: S38 Fig — (A) Representation of liver zinc content comparing individuals with either the AA (n = 11), AG (n = 52) or GG (n = 80) genotypes at rs1047626. (B) Representation of liver zinc content according to the A (n = 74) or G (n = 212) alleles at rs1047626. No statistically significant differences were found despite the observed tendency towards higher zinc concentrations in individuals carrying the derived G-allele. Data are reported as mean ± SE (error bars). Liver zinc content corresponds to the corrected zinc levels previously quantified [23] in the liver samples genotyped here for the rs1047626 polymorphism. (PDF) [file pgen.1010950.s038.pdf]
