# Supplementary material for: Human genetic adaptation related to cellular zinc homeostasis
Source: PLoS Genet. 2023 Sep 25;19(9):e1010950. doi: 10.1371/journal.pgen.1010950 (PMC10553801; doi:10.1371/journal.pgen.1010950)
Supplement: S1 Appendix — (DOCX) [file pgen.1010950.s060.docx]

**S1 Appendix**

**PopHuman genome browser** https://pophuman.uab.cat/?loc=chr4%3A41081001..42990000&tracks=DNA%2Cgene_annotations%2CFst_CHB2YRI_10kb%2CXPEHH_CHB2YRI_10kb%2CFst_CHB2CEU_10kb%2CFst_CEU2YRI_10kb%2CFayWu_H_CEU_100kb%2CFayWu_H_CHB_100kb%2CXPEHH_CEU2YRI_10kb%2CXPEHH_CHB2CEU_10kb&highlight=

**Ensembl genome browser** https://www.ensembl.org/Homo_sapiens/Variation/Population?db=core;r=4:41990160-41991160;v=rs2581434;vdb=variation;vf=91812157 https://www.ensembl.org/Homo_sapiens/Variation/Population?db=core;r=4:42001154-42002154;v=rs1047626;vdb=variation;vf=90883935 https://www.ensembl.org/Homo_sapiens/Variation/Population?db=core;r=4:42002461-42003461;v=rs2581452;vdb=variation;vf=91812411 https://www.ensembl.org/Homo_sapiens/Variation/Population?db=core;r=4:42008367-42009367;v=rs2660319;vdb=variation;vf=91893456 https://www.ensembl.org/Homo_sapiens/Variation/Population?db=core;r=4:42012699-42013699;v=rs1848182;vdb=variation;vf=91328231 https://www.ensembl.org/Homo_sapiens/Variation/Population?db=core;r=4:42016115-42017115;v=rs2581424;vdb=variation;vf=91812026 https://www.ensembl.org/Homo_sapiens/Variation/Population?db=core;r=4:42019947-42020947;v=rs15857;vdb=variation;vf=90289043 https://www.ensembl.org/Homo_sapiens/Variation/Population?db=core;r=4:42027941-42028941;v=rs7439806;vdb=variation;vf=93827974 https://www.ensembl.org/Homo_sapiens/Variation/Population?db=core;r=4:42032677-42033677;v=rs55835604;vdb=variation;vf=98847428 https://www.ensembl.org/Homo_sapiens/Variation/Population?db=core;r=4:42040359-42041359;v=rs12510574;vdb=variation;vf=96046952 https://www.ensembl.org/Homo_sapiens/Variation/Population?db=core;r=4:42054622-42055622;v=rs4861014;vdb=variation;vf=92834899 https://www.ensembl.org/Homo_sapiens/Variation/Population?db=core;r=4:42070955-42071955;v=rs10019356;vdb=variation;vf=94902147 https://www.ensembl.org/Homo_sapiens/Variation/Population?db=core;r=4:42078597-42079597;v=rs7660223;vdb=variation;vf=93923416 https://www.ensembl.org/Homo_sapiens/Variation/Population?db=core;r=4:42086660-42087660;v=rs11051;vdb=variation;vf=90275298 https://www.ensembl.org/Homo_sapiens/Variation/Population?db=core;r=4:42087866-42088866;v=rs11935648;vdb=variation;vf=95766474 https://www.ensembl.org/Homo_sapiens/Variation/Population?db=core;r=4:42088740-42089740;v=rs12511999;vdb=variation;vf=96064268 https://www.ensembl.org/Homo_sapiens/Variation/Population?db=core;r=4:42088974-42089974;v=rs10938178;vdb=variation;vf=95296008 https://www.ensembl.org/Homo_sapiens/Variation/Population?db=core;r=4:42089066-42090066;v=rs12512101;vdb=variation;vf=96065575

**UCSC Genome Browser**

https://genome.ucsc.edu/ (Human (GRCh38/hg38), chr4:41,540,836-42,540,155)

**Vindija33.19 VCF files**

http://cdna.eva.mpg.de/neandertal/Vindija/VCF/Vindija33.19/

**AltaiNeandertal VCF files**

http://cdna.eva.mpg.de/neandertal/Vindija/VCF/Altai/

**Denisova VCF files**

http://cdna.eva.mpg.de/neandertal/Vindija/VCF/Denisova/

**Chagyrskaya-Phalanx VCF files**

http://cdna.eva.mpg.de/neandertal/Chagyrskaya/VCF/

**ARGweaver UCSC Genome browser track** http://compgen.cshl.edu/ARGweaver/introgressionHub/hub.txt

**Recombination maps**

https://github.com/eyherabh/genetic_map_comparisons

**Geography of Genetic Variants Browser**

https://popgen.uchicago.edu/ggv/ (rs1047626, rs4861157)

**ALFRED database**

https://alfred.med.yale.edu/alfred/index.asp (rs1047626)

**GTEX portal**

https://www.gtexportal.org/home/ (rs2581434, rs1047626, rs2581452, rs2660319, rs1848182, rs2581424, rs15857, rs7439806, rs55835604, rs12510574, rs4861014, rs10019356, rs7660223, rs11051, rs11935648, rs12511999, rs10938178, rs12512101, rs4861157)

**GWAS catalog**

https://www.ebi.ac.uk/gwas/ (SLC30A9)

**GWAS atlas**

https://atlas.ctglab.nl/PheWAS (rs1047626)
